# Supplementary material for: Modeling tissue‐relevant Caenorhabditis elegans metabolism at network, pathway, reaction, and metabolite levels
Source: Mol Syst Biol. 2020 Oct 6;16(10):e9649. doi: 10.15252/msb.20209649 (PMC7537831; doi:10.15252/msb.20209649)
Supplement: Supplementary file 1 — Appendix [file MSB-16-e9649-s001.pdf]

APPENDIX

C. elegans tissue-level metabolism at network, pathway, reaction, and metabolite scales

L. Safak Yilmaz[1], Xuhang Li[1], Shivani Nanda[1], Bennett Fox[2], Frank Schroeder[2], and Albertha J.M. Walhout[1]

1 Program in Systems Biology, Program in Molecular Medicine, University of Massachusetts Medical School, Worcester, MA,

2 Boyce Thompson Institute and Department of Chemistry and Chemical Biology, Cornell University, Ithaca, NY

Contents

SUPPLEMENTARY METHODS 1
Updates in *C. elegans* Metabolic Network Reconstruction 1
  Addition of ascaroside biosynthesis 1
  New transport reactions from Recon3D 1
  Additional annotations from ElegCyc 1
  Other modifications and categorization of updates in reconstruction 2
  Quality check of the reconstruction 3
Processing of the Gene Expression Dataset 4
  Determination of absolute thresholds for gene categorization 4
  Relative expression (tissue level enrichment/depletion) analysis 5
Dual-Tissue Model for Data Integration 6
  Conversion of iCEL1314 to a dual-tissue model 6
  Constraint-based flux balance analysis 8
Integration Algorithm 10
  iMAT .... **Error! Bookmark not defined.**
  iMAT++ 12
  Integrations with the dual-tissue model 14
Agreement Between Gene Expression and Optimal Flux Distributions 17
Validation of Predicted Flux Distributions Based on Known Tissue Functions 18
  Calculation of biomass yield 19
Flux Variability Analysis of Tissue-Level Metabolic Network Models 19
Flux Potential Analysis for Quantitative Integration of Expression Data 20
  Gene expression coefficients 22
  Metabolic distance 23
  Calculation of relative flux potentials 24
  Metabolite-level FPA 24
Systematic Analysis of Tissue Function Based on Flux Potentials 25
Summary of Findings with MERGE 26
Robustness and Usability of MERGE 26
  Flux thresholds 26
  Network building algorithms 27
  Distance order 28
  Relative expression analysis 28

Code and model availability....28
SUPPLEMENTARY FIGURES....30
Figure S1....32
Figure S2....33
Figure S3....34
Figure S4....35
SUPPLEMENTARY TABLES....36
Table S1....37
Table S2....38
Table S3....39
SUPPLEMENTARY REFERENCES....40

SUPPLEMENTARY METHODS

Updates in *C. elegans* Metabolic Network Reconstruction

Our previously reconstructed *C. elegans* metabolic network model, iCEL1273(Yilmaz & Walhout, 2016), was manually updated in this study to create iCEL1314 (**Figure 1B**). Details of the updates in the reconstruction are explained below and tabulated in **Table EV1**. The reactions and metabolites of iCEL1314 are shown in **Tables EV2** and **EV3**, respectively.

Addition of ascaroside biosynthesis

Ascaroside biosynthesis was reconstructed based on up-to-date data on ascaroside abundance, synthesis, and breakdown. Although hundreds of different types of ascarosides have been detected in *C. elegans* (Artyukhin *et al*, 2018), many of these compounds are modular derivatives of a relatively small number of ascarosides, including ascr#1, ascr#2, ascr#3, ascr#5, ascr#7, ascr#10, and ascr#18, which together comprise the majority of the ascaroside pool (Ludewig *et al*, 2013). Thus, although key biosynthetic steps remain enigmatic, we reconstructed simplified pathways that form these seven molecules, as shown in **Figure EV1**.

New transport reactions from Recon3D

Little is known about metabolite transport in *C. elegans*. Therefore, transport reactions in iCEL1273 were based on previous human and yeast reconstructions (Schellenberger *et al*, 2010). Since the development of iCEL1273 (Yilmaz & Walhout, 2016), a major modification was made in human reconstruction, resulting in an updated human metabolic model named Recon3D (Brunk *et al*, 2018). We downloaded all human transport reactions of this new model from the Virtual Metabolic Human website (www.vmh.life) and systematically searched all reactions that transported metabolites between extracellular space and cytosol and that could add new transport capabilities to our new reconstruction. Protons were removed from all these reactions to avoid complications with proton balance during ATP synthesis, as was done previously (Yilmaz & Walhout, 2016). If multiple transport reactions were available for the same metabolite in the same direction (*e.g.*, one that cotransports an ion together with transported metabolite, and another that transports the same metabolite alone), and if some of these reactions had more companion molecules than others, then the simplest reaction form was used, also following the previous procedure (Yilmaz & Walhout, 2016). Overall, 121 new transport reactions were incorporated from the human model (**Table EV1**).

Additional annotations from ElegCyc

To further optimize our reconstruction, we evaluated the ElegCyc model, which was developed around the same time as iCEL1273 (Gebauer *et al*, 2016). We downloaded the provided SBML version of ElegCyc and listed all reactions and their gene associations. We specifically searched reactions that did not exist in our model, or that had different gene annotations than our model. Annotations were manually checked using literature and other annotation resources, and evaluated based on our annotation and previous reconstruction criteria (Yilmaz & Walhout, 2016). We excluded reactions that passed our annotation criteria but were not connected to the rest of the metabolic network, as such reactions cannot carry flux under any condition, and therefore, do not contribute to model predictions. However, when multiple disconnected reactions could together be rescued by only one transport, sink, or demand reaction, then these reactions were still considered

for reconstruction, as consistent with our systematic gap-filling approach (Yilmaz & Walhout, 2016). In the end, three new reactions passed our screen: reaction RC01570 associated with gene *upp-1*, RC01951 associated with *K03H1.13*, RC05802 associated with *ppk-3*, and RC03161 associated with *C26D10.4*. Among the four genes associated with these reactions, *upp-1* and *ppk-3* were already part of iCEL1273, but were assigned to other reactions that are maintained in the new model. Thus, a total of four new reactions and two new genes were acquired from ElegCyc. RC01570 and RC01951 did not carry any flux in our model initially. To rescue these reactions, we added SNK0101, a fucose sink that serves as the only source of fucose in the current reconstruction.

Other modifications and categorization of updates in reconstruction

Differences of iCEL1314 with respect to the original model iCEL1273, including the updates mentioned above and others, were divided into 14 main categories (**Table EV1**). These categories and their contents are explained below. When necessary, the details of each change in the model is presented in the "Notes" column of **Table EV1**, or in the "Comments" section of reaction pages in wormflux (http://wormflux.umassmed.edu).

Ascaroside biosynthesis: See above.

FBA: Flux balance analysis (FBA)-based changes in the new model. Includes the removal of reactions that were found to be dependent on flux loops disconnected from the rest of the network, the addition of reactions necessary to connect others introduced to the network, and the inclusion of previously disconnected reactions (Yilmaz & Walhout, 2016) that can now carry flux because of the updates.

Malate-aspartate shuttle: Modifications to establish a proper malate-aspartate shuttle, which was missing in the original reconstruction (Yilmaz & Walhout, 2016).

Manual curation of gene-protein-reaction (GPR) associations: Changes made in GPR associations either based on updates in the annotation resources used (Yilmaz & Walhout, 2016) (mostly in KEGG (Kanehisa *et al*, 2015)) or based on literature search and curation.

Manual curation of protein localization: Re-localization of some reactions between cytosolic and mitochondrial compartments based on manual re-evaluations of the subcellular localization of associated proteins using literature and previous resources (Yilmaz & Walhout, 2016).

Mass balance: Corrections in mass balance of reactions or rebalancing of reactions due to modifications in metabolite formulas.

New biomass reactions: Addition of new biomass reactions to cover all combinations of biomass assembly with respect to the inclusion of collagen, DNA, and reserve metabolites.

Physiological evaluations: Modifications in availability or subcellular locations of metabolites and reactions based on knowledge about *C. elegans* physiology or general animal biology.

Removal of pseudogenes and dead genes: Based on updates in WormBase (Harris *et al*, 2013).

Reversibility: Modifications and corrections in reaction reversibility.

Sphingolipid metabolism: Adjustments in metabolic network due to a modification in sphingolipid composition based on (Witting *et al*, 2018).

Transport updates based on new human model: See above.

Ubiquinone biosynthesis: Corrections in localization and GPR annotations of ubiquinone biosynthesis pathway reactions.

Minor: Changes in metabolite or reaction names, and the necessary insertion or deletion of transport and exchange reactions, as a result of other modifications from the categories above.

Quality check of the reconstruction

The quality of the updated model was tested with MEMOTE standards, which was published when our original manuscript was in review (Lieven *et al*, 2020), with the help of the web tool (*https://memote.io/*). We double-checked the areas where our model seemed to underperform or where the model properties could not be resolved by the web tool, and report our evaluations here.

Stoichiometric consistency: The model scored only 21% in this topic, which we determined to be because of our irregular demand reactions that had metabolites on the products side unlike regular demand reactions. For example, the reaction DMN0073 ((2.0) gtp + (0.324) atp + (2.324) h2o + sectrna --> (2.0) gdp + (0.324) adp + (3.324) h + (2.324) pi + trnasec) represents demand for selenocysteine as this amino acid is missing on the right hand side. As a test, we replaced this reaction with one that includes the missing selenocysteine as a product and another that drains this selenocysteine, which together form a regular demand setting without changing the model capabilities mathematically. This adjustment increased the stoichiometric consistency score to 98%. Removing all irregular demand reactions increased the score to 100%. Since the inconsistency detected by the algorithm has no effect on the performance of iCEL1314, we kept the reactions from the original reconstruction untouched.

Mass and charge balance: Although the model scored high in mass and charge balance, about 3% of the reactions were reported to have either mass or charge balance issues. As was done in the original publication (Yilmaz & Walhout, 2016), we checked mass balance in all reactions and we did not find any reactions out of balance, except for a few reactions like irregular demand reactions with recovered products (see above for the DMN0073 example), which are all meant to be out of mass balance as in regular demand or exchange reactions.

Unbounded fluxes: Eighty reactions in the model can carry infinite flux because of flux cycles (*e.g.*, that between RCC0001 and RMC0001, which represent reversible aconitate hydratase reactions in cytosol and mitochondria, respectively). Since a flux minimization is applied in the end of most applications, these unbounded fluxes are not observed in predicted flux distributions. In addition, we verified by FBA that maximum energy generation capacity of iCEL1314 is zero, when no energy source is provided, indicating that flux cycles do not affect the energy balance of the network.

Blocked reactions: All reactions in iCEL1314 can carry flux except for a total of 94 transport and exchange reactions. These reactions are included in the network so that new reactions with transportable metabolites can be conveniently added to the model (Yilmaz & Walhout, 2016). The web tool found 106 blocked reactions in iCEL1314 including some regular reactions and sinks and demands. We verified that all these extra reactions can actually carry flux when model boundaries are open.

Biomass reactions and growth associated maintenance: iCEL1314 has 8 biomass reactions (BIO0100-BIO0101) and the biomass from any of these reactions is drained by a master biomass reaction BIO0010, which includes ATP degradation to address the

growth associated maintenance (GAM) requirement (Yilmaz & Walhout, 2016). The current MEMOTE algorithm fails to pick GAM in our setting and considered BIO0015 as another biomass production reaction, although this reaction is only a part of the biomass assembly subsystem.

Gene annotations: For easy interpretation, we annotate genes based on gene names used by the worm community in our tables (*e.g.*, **Table EV3**). However, we added KEGG (Kanehisa *et al.*, 2015), Uniprot (UniProt, 2019), and unique WormBase IDs to gene annotations in the official SBML model (see below). Also, the model is linked to KEGG and WomBase through the model website (*http://wormflux.umassmed.edu/*). Although the MEMOTE score of iCEL1314 for gene annotation remains low (41%), these links are sufficient for most applications with a *C. elegans* model.

Processing of the Gene Expression Dataset

Tissue-level consensus expression profiles were downloaded from the reference study (Cao *et al*, 2017). These profiles provide expression of protein-coding genes in seven tissues (**Figure 1D**) as transcripts per million (TPM). We categorized every gene in every tissue as highly, moderately, lowly, or rarely expressed (**Table EV4**) using absolute and relative expression values as explained below. The term “rarely” is used to mean undetected or very close to undetected.

Determination of absolute thresholds for gene categorization

For each gene, TPM values from the seven tissues were first averaged and zeros (*i.e.*, genes undetected in all tissues) were removed. Then, a histogram of the logarithm (base 2) of these average TPM values was generated using 100 bins (**Figure EV2**). Frequency values in this histogram were fitted by a function (F) that represents the superimposition of two other functions as shown in **Equation S1**, where, f_{HES} and f_{LES} stand for the two normal distributions that are used to define the highly expressed and lowly expressed subpopulations of genes (HES and LES, respectively). Each normal distribution is a function of midpoints of TPM bins (x), mean (μ) and standard deviation (σ) of the pertaining subpopulation, and the total number of genes in HES (N_{HES}). N_{total} is the total frequency of the histogram (*i.e.*, the total number of non-zero genes). Since this number is a known constant (N_{total} = 19,432), F was fitted to histogram data to derive the best-fitting values of the remaining five variables of this function as follows: μ_{HES} = 5.00, σ_{HES} = 1.96, μ_{LES} = -1.20, σ_{LES} = 3.13, and N_{HES} = 9,877. With these values, F function fitted the histogram with R^2 > 0.99, thus verifying the assumption that gene expression data consisted of two normally distributed subpopulations. Curve-fitting was achieved using the *curve_fit* function of *optimize* module in *SciPy* library of Python programming language (version 2.7).

$$F(\mu_{HES},\sigma_{HES},\mu_{LES},\sigma_{LES},N_{HES}) = f_{HES}(\mu_{HES},\sigma_{HES},N_{HES})$$
$$+ f_{LES}(\mu_{HES},\sigma_{HES},N_{total} - N_{HES}) \qquad \textbf{S1}$$

We used μ_{HES}, μ_{LES}, and σ_{LES} values to determine absolute thresholds that define rarely (TPM < $2^{\mu_{LES}}$), lowly ($2^{\mu_{LES}}$ ≤ TPM < $2^{\mu_{LES}+\sigma_{LES}}$), and highly (TPM > $2^{\mu_{HES}}$) expressed genes in each tissue, as depicted in **Figure S1** and **Figure EV2**. The remaining genes were initially considered as moderately expressed and further categorized as highly or lowly

expressed based on relative expression analysis as well as μ_{HES} and σ_{HES}, as described next.

Relative expression (tissue level enrichment/depletion) analysis

Expression levels of some of the genes that could not be categorized based on hard thresholds (*i.e.*, moderately expressed genes) still varied greatly between different tissues, indicating that these genes are relatively enriched in some tissues and depleted in others (**Figure EV3**). To capture relative differences in tissue-level expression, we used a heuristic approach and developed a custom algorithm that was trained to produce a convincing output for a test set of more than 200 metabolic genes that were visually inspected. We reevaluated the status of moderately expressed genes (*i.e.*, those with TPM values between 3.8 and 32, **Figure EV2**) with this algorithm. Moderately expressed genes that were found to be relatively highly expressed or relatively lowly expressed were allocated to the highly expressed or lowly expressed category, respectively, but only if their expression was also greater or smaller than a threshold (τ_{rel} = 8.2). This threshold is positioned one standard deviation below the mean of the HES genes (**Figure EV2**, and see above), where HES and LES still overlap significantly. The full description of the algorithm is as follows:

Given a query gene, arrange tissue expression levels in TPM values (x) in ascending order (see **Figure EV3** for examples).

$$x_1 \leq x_2, \ldots, x_{k-1} \leq x_k, \ldots, x_6 \leq x_7$$

First, determine relatively high expression values. From position 2 to 7 (k = 2 to 7), check:

$$x_k > \tau_{rel} \qquad \textbf{S2}$$

$$\frac{x_k}{x_{k-1}} > FC_k^{high} \qquad \textbf{S3}$$

where, FC_k^{high} is 4.0 for k = 2 and 1.5 for all other positions.

If inequalities **S2** and **S3** are both true, then label expressions in k to 7 as "high", and those in 1 to k-1 as "not high" (if expression in a tissue is previously labeled, the existing label is not overwritten; all labels are maintained to make a final decision in the end, see below).

Next, determine relatively low expression values. From position 2 to 7 (k = 2 to 7), check inequality **S2** and

$$\frac{x_k}{x_{k-1}} > FC_k^{low} \qquad \textbf{S4}$$

where, FC_k^{low} is 4.0 for k = 7 and 1.5 for all other positions.

If inequalities **S2** and **S4** are both true, then label expressions in 1 to k-1 as "low" and those in k to 7 as "not low".

Finally, move moderate expression values that are greater than τ_{rel} and labeled as "high", but not as "low" or "not high", to high category; and move moderate expression values that are smaller than τ_{rel} and labeled as "low", but not as "high" or "not low", to low category.

The stringency of this algorithm was optimized by changing the fold change requirements. The reason for having a conservative 4-fold change requirement at the highest and lowest points in the expression profile is to discourage the labeling of six tissue expressions altogether as high or low, when this decision is solely based on the expression reported for the remaining single tissue. The requirement in statement **S2** is included to ignore fold changes when the two numbers compared in **S3** and **S4** are both small, as changes in the low scale may not be meaningful. Overall, this algorithm captures significant leaps in the TPM profiles of some variably expressed genes, thereby defining groups of tissues with consistently and relatively high or low expression levels (**Figure EV3**). If expression in a group of tissues is both relatively higher than that in one other group and lower than that in yet another, then no high or low decision is made for this group in the end (*e.g.*, see expression of *E01A2.1* in neurons and hypodermis, **Figure EV3**). On average, the ratio of moderately expressed genes (in the original set) converted to highly and lowly expressed genes by this algorithm was 11 +/- 7% and 22 +/- 3%, respectively (average +/- standard deviation for the seven tissues).

Dual-Tissue Model for Data Integration

Conversion of iCEL1314 to a dual-tissue model

Reactions of the generic metabolic network model iCEL1314 were compartmentalized to develop a dual-tissue model with four compartments (**Figure 2A**, **Table EV5**). These compartments and the different sets of reactions included in each compartment are as follows (see **Table EV5** and **Table S1**):

Lumen (*L*): This compartment accommodates exchange reactions associated with bacterial molecules or selected side nutrients. We define side nutrients as all metabolites of iCEL1314 that can be acquired by existing transport or sink reactions, but that are not part of, or are quantitatively underestimated in, the assumed bacterial biomass composition. This definition excludes metabolites that can be acquired but are highly unlikely to be a part of the standard diet of *C. elegans* (*e.g.*, sphingomyelin, acetylcholine, histones, *etc*.). There are 243 side nutrients in total (**Table EV3**), of which, 229 can be imported in from the environment (*i.e.*, associated with a transport reaction rather than a sink reaction). All importable side nutrients, as well as the metabolite that represents bacterial food (BAC), are brought from environment into the lumen with unidirectional exchange reactions, which we call uptake reactions. Products of degradation reactions of bacterial molecules can also be secreted out to the environment from the lumen if they are not used by the model during flux balance analysis, which represents the wasting of bacterial biomass. Also included in lumen are extracellular reactions, other than transport

or exchange, which convert metabolites within the extracellular space. Reactions that degrade bacterial biomass outside cytoplasm are included in this set.

Intestine (*I*): All intracellular reactions (*i.e.*, those confined to cytosol and mitochondria) are included in the intestine compartment. The only difference from the internal reactions of iCEL1314 is in sinks, which are reversible reactions that can drain or provide specific metabolites. In the dual model, these reactions are represented as two separate irreversible reactions, one for the forward and the other for the reverse direction. The reverse reactions act as uptake reactions, as the ones in lumen, except that the metabolite taken up is directly brought into the cell, as the reaction represents the utilization of stored resources within the cell. Most of these metabolites are side nutrients (see above), and three of them are storage molecules including glycogen, trehalose, and triacylglycerides (TAG) (see below).

Other tissue (*X*): As with intestine, this compartment accommodates the internal reactions of the generic model with modifications in sink reactions. However, any reaction that cannot carry flux due to lack of bacterial molecules (due to the absence of a connection between other tissue and lumen; **Figure 2A**) is excluded.

Extracellular space (*E*): This compartment includes unidirectional exchange reactions that can secrete waste products from intestine or other tissue compartments to the environment, as well as specific reversible exchange reactions for oxygen, water, phosphorus, and protons, so that these molecules can be both freely secreted to and taken up from the environment. In addition, as with the lumen compartment, extracellular reactions in the model that convert metabolites within the extracellular space are included in the *E* compartment, except for those which degrade bacterial molecules.

In addition to the compartment-specific reactions described above, available transport reactions in iCEL1314 are used to exchange transportable metabolites between lumen and intestine, intestine and extracellular space, and other tissue and extracellular space. All metabolites in a compartment are labeled by the letter designating that compartment in the end of the metabolite name (*I*, *X*, *L*, or *E*, see above). Compartmentalized labeling of metabolites is mathematically sufficient to carry out FBA with the dual-tissue model, as reaction stoichiometry is described based on metabolite names. However, reactions and genes must also be labeled after the compartments for the sake of the interpretability of integration algorithms, specific constraints, and tissue-level results. All compartment-specific reactions are labeled by the same four letters representing different compartments. Transport reactions are assigned to one of the two compartments they are associated with. Transports between *L* and *I* belong to *L*, those between *I* and *E* belong to *I*, and those between *X* and *E* belong to *X*. Among transports that belong to *I*, originally irreversible import reactions were converted to reversible transports to allow the delivery of importable nutrients from intestine to other tissues. An exception for reaction labeling is the extracellular reactions in the *L* and *E* compartments; these reactions are assigned to *I* and *X* compartments, respectively. Genes in the dual model are also labeled by the compartment suffixes, the same as the reactions they are associated with, except that those associated with transport reactions functioning between lumen and intestine are assigned to intestine. The entire dual model with reactions and genes is presented in **Table EV5**, and the key metrics that characterize the model compartments are in **Table S1**. Overall, the dual tissue model has 4506 reactions and 2865 metabolites, including those in **Table S1** and two additional artificial metabolites that help flux balance constraints, as will be described below.

Constraint-based flux balance analysis

All simulations with the dual-tissue model were performed using FBA (Raman & Chandra, 2009) with extensions that address specific goals in different types of applications. Two basic sets of FBA constraints were used in all simulations, as shown with **Equations S5** and **S6**. **Equation S5** establishes a steady-state mass balance for every metabolite in the network and is shown both as a system of equations and in the form of matrix notation. In this equation, flux in a reaction *j* is represented by v_j, and s_{ij} stands for the stoichiometric coefficient of a metabolite *i* in that reaction. M_{all} and R_{all} designate the sets of metabolites and reactions in the entire network, respectively. The flux is a positive value if the pertaining reaction is proceeding in the forward direction, and a negative value if it is proceeding backwards. The direction of a reaction is defined with respect to the order of reactants and products in the reaction, from left to right, as given in model tables (**Tables EV2** and **EV5**). Stoichiometric coefficients are negative for reactants and positive for products, as reactants are consumed and products are produced by a positive flux. For each metabolite, the net production rate is zero at steady state, hence the 0 value on the right hand side. In the matrix notation, $\boldsymbol{S}$ is the matrix of s_{ij} values, $\boldsymbol{v}$ is the vector of all reaction fluxes, and **0** is the zero vector indicating metabolite production rates at steady state. **Equation S6** defines the lower (LB) and upper (UB) boundaries of flux for each reaction, mostly to set reaction reversibility rules. Typical (LB, UB) pairs used are (-1000, 1000) for a reversible reaction, (0, 1000) for a forward-only reaction, and (-1000, 0) for a backward-only reaction (applies to uptake reactions only), as 1000 is sufficiently large to represent infinite flux in our simulations. Exceptional (LB, UB) pairs for specific reactions will be indicated below.

$$\sum_{j \in R_{all}} s_{ij} v_j = 0; i \in M_{all} \qquad \textbf{S5}$$

$$\boldsymbol{S} \cdot \boldsymbol{v} = \boldsymbol{0}$$

$$v_j^{LB} \leq v_j \leq v_j^{UB} \qquad \textbf{S6}$$

<u>Constraining side and storage metabolites</u>

To limit the usage of side and storage metabolites as compared to ingested bacterial biomass, we developed a system of reactions and linear constraints that can be applied during FBA. Given a set of metabolites that can be consumed (M_{uptake}), the uptake reaction of each metabolite is designed as shown in **Equation S7**, where m_k, *A*, and c_k designate the *k*'th metabolite in M_{uptake}, an artificial metabolite commonly used in all uptake reactions for M_{uptake}, and the coefficient of this artificial metabolite, respectively. The coefficient of *A* is calculated as a fraction of the molecular weight of the metabolite consumed (W_k) (**Equation S8**). The division by 100 was introduced to avoid relatively large coefficients in uptake reactions as compared to others, which would create a numerically unbalanced stoichiometry matrix ($\boldsymbol{S}$, **Equation S5**). Thus, the amount of *A* introduced, which equals the flux of the uptake reaction (v_k, **Equation S7**) multiplied by c_k, is proportional to the weight of metabolite m_k consumed by the model (in mg/dW/h, where dW stands for dry weight of *C. elegans* biomass in grams) as all fluxes are in molar units (mmol/dW/h) and the molecular weights are in mg/mmol (equal to g/mol). Total *A* introduced to the system from the uptake of all metabolites in the set M_{uptake} is drained by a single reaction (**Equation S9**), the flux of which (v_A) must equal the total production of *A* from all relevant

uptake reactions by mass balance. This relationship is shown in **Equation S10**, where the negative sign is due to the fact that all uptake fluxes are negative whilst the flux of the reaction that drains *A* (**Equation S9**) is positive.

$$m_k + c_k A \overset{v_k}{\leftarrow} \quad \textbf{S7}$$

$$c_k = \frac{W_k}{100} \quad \textbf{S8}$$

$$A \overset{v_A}{\rightarrow} \quad \textbf{S9}$$

$$v_A = \frac{-1}{100} \sum_{k \in M_{uptake}} W_k v_k \quad \textbf{S10}$$

It is straightforward to constrain v_A, and hence the total metabolite uptake for metabolites in M_{uptake}, to be a fraction of the bacterial ingestion on a weight/weight basis. To enforce such a fractional limitation, we use a linear constraint as shown in **Equation S11**, where W_{BAC} stands for the weight of bacterial nutrients ingested when the exchange reaction that brings in bacteria to the system (EXC0050) has a unit flux, v_{BAC} is the flux of this reaction, and α is the weight fraction used (*e.g.*, α =0.01 when all nutrients in the set M_{uptake} combined is allowed to be at most 1% of ingested bacteria by weight). The bacterial exchange reaction EXC0050 is designed to bring in 1 g of bacterial mass per unit flux (Yilmaz & Walhout, 2016) , but since ~3.4% of the assumed composition of bacterial biomass includes soluble molecules unaccounted for as well as inorganic matter, the value of W_{BAC} was set at 966.4 mg. The negative sign in **Equation S11** is due to the fact that bacterial ingestion flux is always negative. In addition to limiting the uptake of all metabolites in a set, we can also limit the uptake of each individual metabolite using the type of constraint shown in **Equation S12**, where β is the weight fraction factor like α. However, the individual weight factor β must be smaller than α for it to be functional, as v_A covers all metabolites in M_{uptake}.

$$v_A \leq -\alpha \frac{W_{BAC} v_{BAC}}{100} \quad \textbf{S11}$$

$$c_k v_k \leq -\beta \frac{W_{BAC} v_{BAC}}{100} \quad \textbf{S12}$$

In the dual-tissue model, we used the system of reactions and constraints in **Equations S7-S12** to limit the uptake rates of two sets of metabolites: side metabolites and storage metabolites (**Table EV3**). The artificial metabolites (*A*) used for this purpose are sideMet and storageMet, respectively (**Table EV5**, uptake reactions starting with “UP”). The weight factors α and β varied in different contexts as will be described below.

Objective function and other constraints

During FBA, a particular objective is achieved while a set of required linear constraints, such as those described above, are all satisfied. In regular applications of FBA, this objective is the maximization of a sum of reaction fluxes, as shown in **Equation S13**, where w indicates reaction weight. However, the objective function can take different forms in flux analysis methods that are extensions of FBA. We used problem-specific objectives and (when necessary) additional constraints, which will be described in the sections that follow.

$$obj.fnc. = \max\left(\sum_{i\in R_{all}} w_i v_i\right) \qquad \textbf{S13}$$

Integration Algorithm

To integrate gene expression data with the dual-tissue model, we used mixed integer linear programming (MILP), building on a method that was originally proposed by Shlomi et al. (Shlomi *et al*, 2008) and was later incorporated into an integration tool called iMAT (Zur *et al*, 2010). We optimized iMAT algorithm to develop iMAT++. These two algorithms are described next. Then, we will describe how the integration algorithms are applied to the dual-tissue model specifically (**Figure 2A**).

iMAT

We used the original algorithm (Shlomi *et al.*, 2008) with minor modifications and a flux minimization in the end (Yilmaz & Walhout, 2016). First, the gene categorization in **Figure 1E** was modified by merging rarely and lowly expressed genes into one group, named lowly expressed, as the original algorithm does not include a rarely expressed category. Then, the information in highly and lowly expressed gene sets was converted to presumed flux states of a subset of reactions based on GPR associations. The presumed flux states are ON or OFF, defining two sets of reactions, named R_{on} and R_{off}, which are forced to carry flux and to have no flux, respectively. To determine if a reaction belongs to R_{off}, all lowly expressed genes in the GPR of this reaction were replaced by 0 (or False) and other genes by 1 (or True). Together with the AND and OR connections in the GPR (Jensen *et al*, 2011), these replacements convert the GPR into a logical expression that can be evaluated to obtain an overall result as 1 (True) or 0 (False). If the result is 0, the queried reaction is dependent on the lowly expressed genes it is associated with, and is therefore added to the R_{off} set. Reactions in R_{on} were simply determined as those associated with highly expressed genes except those which were already assigned to R_{off}. This approach to determine R_{on} included the only modification from the original algorithm with regard to reaction categorization. If a reaction were dependent on both highly and moderately expressed genes (*e.g.*, a reaction with a GPR of A & B, where A and B are highly and moderately expressed genes, respectively), then the original algorithm would not include this reaction in R_{on}, but we did so in this study to avoid losing information in high expression because of the inconclusive input from moderate expression levels. Reactions for which no state could be determined, either because they are not associated with any gene or because logical operators of their GPRs do not allow an ON or OFF call, form a third set, which is left free in terms of flux state during the integration.

After determining R_{off} and R_{on}, MILP was established using specific constraints (Shlomi *et al.*, 2008). To impose no flux on an OFF reaction, the type of constraint shown in

Equation S14 is applied. The newly introduced variable in this inequality is *y*, which indicates a binary integer (0 or 1). Making the value of this variable 1 converts both boundaries in the inequality to 0, thus constraining the flux to zero value. To force an ON reaction to carry at least a minimum amount of flux (ε, see below for numerical values of this threshold), constraints described in **Equations S15** and **S16** are used for the forward (*f*) and reverse (*r*) directions of reactions (when either one is applicable), respectively. Thus, a binary integer (*y*) of value 1 (**Equation S15**) dictates a flux larger than or equal to a threshold for the forward direction, and less than or equal to the negative of a threshold for the reverse direction (**Equation S16**). For reversible reactions, binary integer of only one direction can be 1, as the flux is zero in at least one direction. For irreversible reactions, only one of these two constraints is applicable.

$$v_i^{LB}(1-y_i) \le v_i \le v_i^{UB}(1-y_i); y_i \in \{0,1\}, i \in R_{off} \quad \textbf{S14}$$

$$v_i \ge v_i^{LB} - y_i^f\left(v_i^{LB} - \varepsilon_i^f\right); y_i^f \in \{0,1\}, i \in R_{on} \quad \textbf{S15}$$

$$v_i \le v_i^{UB} - y_i^r\left(v_i^{UB} + \varepsilon_i^r\right); y_i^r \in \{0,1\}, i \in R_{on} \quad \textbf{S16}$$

During an integration process, it is generally not possible to use *y* variables in **Equations S14-S16** such that all reactions in R_{on} carry flux, while none of those in R_{off} do. This is mainly due to conflicts between R_{on} and R_{off} (*e.g.*, when there are ON and OFF reactions in the same linear pathway). However, the optimization goal of integration is to simultaneously maximize the number of active reactions in R_{on} and inactive reactions in R_{off}. Hence, the sum of *y* variables (Z_{fit}) is maximized as the objective function used in MILP integration (**Equations S17** and **S18**).

$$Z_{fit} = \sum_{i \in R_{on}} (y_i^f + y_i^r) + \sum_{i \in R_{off}} y_i \quad \textbf{S17}$$

$$Z_{fit}^{max} = \max\left(Z_{fit}\right) \quad \textbf{S18}$$

Maximization of the objective in **Equation S18**, while constraints in **Equations S5**, **S6**, and **S14-S16** hold, provides a flux solution that best fits the R_{on} and R_{off} categorization in the network. However, this solution is typically not unique and alternative flux distributions can reach the same objective. Among the alternatives, we derived a solution that minimizes total flux (Z_{total}) using a new objective, while the maximized objective from the previous step was still satisfied using an additional constraint (**Equation S19**) (Yilmaz & Walhout, 2016). Minimizations as those in **Equation S19** were achieved in this study by linearizing the absolute variables (*i.e.*, fluxes, $|v_i|$) using helper variables and additional constraints during linear programming (Sajitz-Hermstein & Nikoloski, 2016), which is possible for minimization problems but not for maximization problems.

$$Z_{total}^{min} = \min\left(\sum_{i}^{N_{rxn}} |v_i|\right) \quad s.t. \;\; Z_{fit} = Z_{fit}^{max} \quad \textbf{S19}$$

iMAT++

We optimized iMAT to develop iMAT++, which includes two major steps (**Figure 2B**). The initial step of iMAT++ finds a preliminary flux distribution (PFD) and is different from iMAT in two ways. The first difference is in the fitting of flux to highly expressed genes. While iMAT is reaction-centered, iMAT++ takes a gene-centered approach, wherein the set of genes that are highly expressed (**Figure 1E**) and associated with at least one reaction in R_{on} (see above) is first determined. This set will be referred to as G_{on}. For each gene in G_{on}, an integer variable (z) is created, subject to the constraints in **Equations S20** and **S21**, where y indicates a binary variable that defines the flux state of a reaction in one direction as explained above, and R_i represents the set of reactions associated with gene i. The first constraint (**Equation S20**) states that the maximum value that the gene integer can take is the number of reactions (in R_{on}) that carry flux and are associated with the gene in question (once again, at most one of y^f and y^r can be 1 for a given reaction, as the reaction can be active in only one direction). However, because of the second constraint (**Equation S21**), this value can never be greater than 1. It follows that, if none of the R_{on} reactions associated with gene i carries flux, then z_i is zero, which indicates that the gene is inactive. Conversely, if at least one of these reactions carry flux, then z_i can take the value of 1, which indicates that the gene is in an active state. In the gene-centered approach, the goal about highly expressed genes is to maximize the number of active genes rather than reactions. Therefore, the integer sum used in the fitting objective function of the reaction centered approach (**Equations S17**) is replaced by that shown in **Equation S22**, while maximization the same as in **Equations S18**.

$$z_i \leq \sum_{k \in (R_i \cap R_{on})} (y_k^f + y_k^r); i \in G_{on} \qquad \textbf{S20}$$

$$z_i \leq 1;\ i \in G_{on} \qquad \textbf{S21}$$

$$Z_{fit} = \sum_{i \in G_{on}} z_i + \sum_{i \in R_{off}} y_i \qquad \textbf{S22}$$

The second difference of iMAT++ compared to iMAT concerns the integration of lowly expressed genes. Different from iMAT, R_{off} in **Equation S22** is the inactive reaction set defined based on associations with rarely expressed genes alone instead of both rarely and lowly expressed genes combined. Lowly expressed genes are a subject of flux minimization. Multiple flux minimization steps exist in iMAT++ (**Figure 2B**) and the first one is applied to a set of reactions dependent on lowly or rarely expressed genes, referred to as R_{low}. This set of reactions was generated following the same approach that produced R_{off} (in iMAT++), except that both lowly and rarely expressed genes are eliminated in logical GPR expressions instead of just rarely expressed genes (R_{off} is a subset of R_{low}). Then, while the maximized objective from **Equations S22** and **S18** is satisfied, the total flux in R_{low} (Z_{low}) is minimized using **Equations S23** and **S24**. Thus flux in reactions dependent on lowly expressed genes is repressed by flux minimization, while the flux in reactions dependent on rarely expressed genes is repressed both by discrete forcing and by flux minimization. Next, while the minimized sum for R_{low} is also held within a tolerance window, total flux in the entire network is minimized as shown in **Equations S25** and **S26**,

to reach the flux distribution called PFD. We set the tolerance variable δ_{low} in this equation at a small value of 1E-5, so that total flux in R_{low} remains close to the minimized value from **Equation S24** in the solution.

$$Z_{low} = \sum_{i \in R_{low}} |v_i| \tag{S23}$$

$$Z_{low}^{min} = \min(Z_{low}) \quad s.t. \quad Z_{fit} = Z_{fit}^{max} \tag{S24}$$

$$Z_{total} = \sum_{i \in R_{all}} |v_i| \tag{S25}$$

$$Z_{total}^{min} = \min(Z_{total}) \quad s.t. \quad Z_{fit} = Z_{fit}^{max}, Z_{low} \leq Z_{low}^{min} + \delta_{low} \tag{S26}$$

A drawback of PFD from iMAT++ is the existence of reactions that are associated with only highly expressed genes, but that do not carry any flux although all of their substrates are active, *i.e.*, used by flux-carrying reactions of the PFD (substrates are only the reactants for irreversible reactions, and either reactants or products for reversible ones). We call such reactions “latent reactions”, as their flux potential is missed by the integration. To address latent reactions, active metabolites in the network are first determined based on a flux threshold. Any metabolite that has a total flux (*i.e.*, the product sum of coefficients and fluxes of reactions that use the metabolite) greater than this threshold is considered active. We used a threshold of 1E-5, which indicates a small but significant flux in the scale of our flux distributions and with respect to the solver parameters used (**Table S2**). The set of no-flux reactions that are associated with only highly expressed genes is also determined. Then, reactions in this set that would convert only active metabolites, if they carried flux, form the set of latent reactions (R_{latent}). Next, while the fitting quality of PFD is maintained, these latent reactions are activated as much as possible, using the objective function described by **Equations S27** and **S28**. Not all latent reactions can be forced to carry flux due to conflicts with rarely and lowly expressed genes as would be captured by the constraints on Z_{fit}, and Z_{low}, respectively. The reason for placing an upper limit on Z_{total} (**Equation S25**) rather than setting this variable free is because, in rare cases, forcing flux in a latent reaction brings an unreasonably large flux cost to the network. The upper limit is defined by the minimization in **Equation S26** plus a tolerance (Δ_{total}), which can be set at a large value, unlike with δ_{low} (see above), as the purpose here is to prevent an extreme case from distorting the flux distribution. We used a Δ_{total} value that is equal to 5% of the total minimized flux (Δ_{total} =0.05Z_{total}^{min}). Significantly lower values increased the number of latent reactions unaddressed, while similar values such as 10% did not change results.

$$Z_{latent} = \sum_{i \in R_{latent}} (y_i^f + y_i^r) \tag{S27}$$

$$Z_{latent}^{max} = \max(Z_{latent}) \quad s.t. \\ Z_{fit} = Z_{fit}^{max}, Z_{low} \leq Z_{low}^{min} + \delta_{low}, Z_{total} \leq Z_{total}^{min} + \Delta_{total} \tag{S28}$$

After maximizing the number of latent reactions by **Equation S28**, total flux in the network is minimized again using **Equation S29**, where Z_{total} follows from **Equation S25**, to reach the new flux distribution with reactions in R_{latent} addressed as much as possible. However, because the flux distribution has changed, new latent reactions are likely to occur. Thus, the set R_{latent} is updated based on the active metabolites of the current flux distribution, and the steps in **Equations S27-S29** are repeated iteratively, until no new latent reactions are introduced. iMAT++ algorithm finishes when this iterative loop is done, and the last minimization of total flux (**Equation S29**) yields the optimal flux distribution (OFD) (**Figure 2B**). We provide code for the generic use of iMAT++ as well as that specifically developed for the *C. elegans* dual-tissue model (see Code and Model Availability section below).

$$Z_{total}^{min} = \min(Z_{total}) \quad s.t. \\ Z_{fit} = Z_{fit}^{max}, Z_{low} \leq Z_{low}^{min} + \delta_{low}, Z_{latent} = Z_{latent}^{max} \qquad \textbf{S29}$$

Integrations with the dual-tissue model

The structure of the dual-tissue model allows the integration of gene expression data for one tissue at a time, with integration of gene expression data for intestine left to the end (**Figure 2A**). Categorization of genes in the intestine (*I*) compartment (*i.e.*, genes labeled with *I* in the end, **Table EV5**) and genes in the *X* compartment (*i.e.*, genes labeled with *X* in the end) were based on the categorization of intestine genes and the other tissue genes, respectively (**Figure 1E**). Input gene and reaction sets used in integration, namely, G_{on}, R_{on}, R_{off} and R_{low}, were determined for each compartment accordingly. First, integrations for the six non-intestinal tissues were carried out one by one. During these integrations, R_{on} and G_{on} sets for the intestine were not used, as the objective was not to get the best-fitting flux distribution for the intestine. However, R_{off} and R_{low} sets included both intestine and other tissue reactions. The reason for including intestine reactions is to avoid the free use of the metabolic functions of lowly or rarely expressed genes inside the intestine compartment for supporting the other tissue, which is actually being integrated.

In the second step (**Figure 2A**), intestinal gene expression data was integrated in the intestine compartment using reaction and gene sets for the intestine only; gene and reaction sets used in the integration of other tissues are irrelevant in this stage. However, the overall flux of material to and from the other tissue compartment needs to be taken into account during the integration for the intestine, as a significant function of this tissue is to support the metabolic activity in the other tissues, which is represented by integrated flux distributions from the first step. Cumulative flux demands of the other tissues can be addressed in the simplest way by taking a sum of the six integrated flux distributions of the *X* compartment, thus obtaining a flux sum for each *X* reaction, and then constraining the flux in each of these reactions to the calculated sum. However, this method creates too many unnecessary constraints, thereby slowing down the rate of computation and causing numerical stability problems. A mathematically equivalent and computationally more efficient approach is to replace the entire *X* compartment with a set of reactions that represent the usage of extracellular metabolites only, as the effect of fluxes in the *X* compartment on the *I* compartment is conveyed through these metabolites. Thus, during the integration for the intestine, we reduced reactions of the *X* compartment to artificial exchange reactions in the form shown in **Equation S30** for the *k*'th metabolite (m_k) in the set of extracellular metabolites (M_E). The cumulative flux of this reaction was calculated

using **Equation S31**, based on the integrated flux distribution of the six non-intestinal tissues (T_6). The inner summation in **Equation S31** calculates total flux through m_k for the *i*'th tissue in T_6. Here, s_{kj} indicates the stoichiometric coefficient of the metabolite *k* in a reaction *j* from the set of reactions in *X* compartment represented by R_X (in most cases only transport reactions are relevant since extracellular metabolites are transported between *E* and *X* compartments). The weight factor, w_i, represents the relative contribution of a tissue to total flux, as will be explained below. Finally, the flux of each exchange reaction (v_k) was constrained by the calculated sum (**Equation S32**).

$$m_k \overset{v_k}{\leftrightarrow} \quad \textbf{S30}$$

$$v_k^{sum} = \sum_{i \in T_6} \sum_{j \in R_X} s_{kj} w_i v_{ij}; k \in M_E \quad \textbf{S31}$$

$$v_k^{sum} \leq v_k \leq v_k^{sum} \quad \textbf{S32}$$

During the calculations for overall flux demands of non-intestinal tissues, artificial metabolites that were used to limit the consumption of side and storage metabolites, *i.e.*, sideMet and storageMet, respectively (see above in the subsection Constraint-based flux balance analysis / Constraining side and storage metabolites), were included in the extracellular metabolite set M_E. Reactions in R_X (**Equation S31**) that produce these metabolites address the usage of intracellular side and storage metabolites from sink reactions and thus reduce the allowed production of sideMet and storageMet (and therefore the total uptake of side and storage metabolites, respectively) during the integration with the intestine. Importantly, since all metabolic activity is considered in the intestine integration step, the integrated flux distribution from this step determines the overall input (bacteria, side metabolites, oxygen, *etc.*) and output (waste products like carbon dioxide) of the animal's metabolism, in addition to the specifications of the intestine metabolism. In what follows, we describe some parameters used in dual-tissue model integrations in further detail.

<u>Determination of flux thresholds</u>

The definition of a flux-carrying reaction is based on a flux threshold (ε) according to **Equations S15** and **S16**. The original study that developed the iMAT algorithm (Shlomi *et al.*, 2008) proposed the usage of a uniform ε value throughout the metabolic network. Although we used a standard ε of 0.01 for >90% of the gene-associated reactions in the dual model, we modified the thresholds of the remaining reactions based on flux variability analysis (FVA) (Mahadevan *et al*, 2002). We first defined a reference nutritional state for simulations by constraining the bacterial ingestion to 1.0 flux unit or less, overall uptake ratios of both side and storage metabolites to 0.01 or less (*i.e.*, α=0.01, **Equation S11**), and the uptake ratio of individual side metabolites to 0.001 or less (*i.e.*, β=0.001, **Equation S12**). Under these nutritional conditions, we calculated the maximum flux each reaction can take in the directions allowed (forward or reverse). This calculation was done using the objective function in **Equation S13**. For the forward direction, the weight (*w*) of the queried reaction was set at 1 and that of all other reactions in the network at 0. These numbers were -1 and 0 for the reverse direction, respectively. The magnitudes of maximum attainable flux in the forward and reverse directions were defined by the maximized objective function value and by the negative of this value, respectively. Then

the obtained maximum value was divided by 2. If the result was greater than the standard ε value (0.01), then no modification was made, but if not, then this result was used as the modified threshold in that direction, so that the reaction would not be overloaded during the integration. A zero threshold indicated that the reaction could not carry flux in the tested direction, and therefore was irrelevant for integration. Zeros were arbitrarily replaced by the standard value of 0.01 to avoid numerical issues. Only the valid direction was evaluated for irreversible reactions. Thus, each gene-associated functional reaction in the network was assigned a flux threshold that is at most one half of its flux-carrying capacity, which prevents overloading of individual reactions during integration.

Optimization of nutritional conditions

The uptake ratios of side metabolites with respect to bacteria (the α and β values in **Equations S11** and **S12**, respectively) need to be optimized to ensure a bacteria-dominated diet. When large ratios are allowed, then easier access to side metabolites of choice for the model minimizes the usage of bacterial biomass. However, too small ratios make critical side nutrients limiting, and bacterial ingestion increases remarkably to uptake these nutrients in adequate amounts, but most of this bacterial biomass is then wasted (defecated from lumen) as it is needed only for allowing more uptake of side metabolites. We therefore tried different α and β values during the integration with iMAT++ and calculated the relative amount of wasted bacterial biomass in the final OFD from the integration of intestine data. Since the cumulative flux of other tissue compartment (**Figure 2A**) is accounted for, OFD from this step describes the overall metabolic activity predicted for L2 animals. Based on the plot of the waste against different uptake ratios for total and individual side metabolites (**Figure S3A**), we decided to use a total uptake ratio of 2% (α=0.02, **Equation S11**) and an individual uptake ratio of 0.5% (β=0.005, **Equation S12**) for side metabolites. On the other side, storage metabolites were limited only by an arbitrary overall uptake ratio at 1% (α =0.01) as the integration results were not sensitive to this number unlike side metabolites. These constraints provided a bacteria-dominant diet with sufficient side and storage metabolites to best fit flux distribution to gene expression data.

Determination of non-growth associated maintenance ATP requirements

The standard non-growth associated maintenance ATP requirement (NGAM) in the original iCEL model (Yilmaz & Walhout, 2016) was set at 1.0 mmol/g dW/h, which indicates the constrained flux of a reaction that converts ATP to ADP (RCC0005, **Table EV2**). In the dual-tissue model, this reaction is represented in both intestine and other tissue compartments, with RCC0005_I and RCC0005_X, respectively (**Table EV5**). The original flux value of 1.0 was based on estimated values of observed bacterial ingestion and growth during FBA (Yilmaz & Walhout, 2016). In this study, we arbitrarily targeted a bacterial ingestion value on the order of 1.0 (bacterial biomass/worm biomass/h) and scaled our flux thresholds accordingly (see above). After integrations using the iMAT++ algorithm and the optimization of nutritional conditions in the previous section, the model ended up using ~2.0 units of bacterial biomass, which is approximately twenty times larger than the original simulation values. To increase the NGAM flux nearly proportionally and divide between the *I* and *X* compartments of the dual-tissue model, we used a lower boundary limit of 10 mmol/g dW/h in both RCC0005_I and RCC0005_X reactions. Since there is always a given constraint in these reactions during integration, this adjustment was done iteratively during the optimization of nutritional conditions.

Determination of tissue weight factors

The summation of non-intestinal tissue flux distributions (**Equation S31**) primarily aims to estimate the combined metabolic needs of these tissues that is to be supplied by the intestine during the integration of intestine expression data (**Figure 2A**). Direct addition of fluxes from different tissues is problematic because fluxes represent values normalized to tissue mass (flux unit is mmol/g dW/h), which is certain to vary significantly across the different tissues (http://wormatlas.org). For instance, if glia excretes a metabolite and the muscle consumes the same metabolite, and if the fluxes representing this export and import are the same in the integrated flux distributions of these two tissues, then overall effect on the intestine must be a net consumption from the two, as the muscle is a larger tissue than glia. While we do not know actual mass of tissues in *C. elegans*, we can approximate relative mass using proxies and reasonable assumptions. Thus, we weighed non-intestinal tissues by a factor (*w*, **Equation S31**) that represents a first approximation to relative tissue mass and activity. To calculate this factor, we used median unique molecular identifier (UMI) counts provided for the single cells whose transcriptomes were aggregated to make tissue transcriptomic profiles (Cao *et al.*, 2017). UMI indicates the size of the mRNA pool, thereby serving as a proxy for cell mass and activity. Our approximation is formulated in **Equation 33**, where, T_6 indicates the set of non-intestinal tissues and C_i represents one of the single cells that were used in the determination of the transcriptomic profile of tissue *i*. Thus, the weight of a tissue is defined as the ratio of UMIs collected from that tissue to overall UMI pool. Since UMIs only provide a rough approximation, we took the logarithm of UMI numbers before summation, so that the data was compressed and large variation of weights was thus avoided unless such variations were supported by clear differences in UMI. Tissue weights calculated this way yielded a profile (**Figure EV4A**) that is consistent with our general understanding of *C. elegans* physiology for the most part (*e.g.*, muscle is the biggest T_6 tissue, whereas glia and pharynx are small, in agreement with **Figure EV4A**). We did not use an additional weight factor anywhere to address the relative mass of the intestine, which is mathematically equivalent to assuming that intestine is equal in mass to all other tissues combined. Since the intestine accounts for roughly a third of *C. elegans* body mass and is believed to be the main organ involved in metabolism (McGhee, 2007), we took this assumption as a reasonable approximation for semi-quantitative modeling and refrained from further adjustments.

$$w_i = \frac{\sum_{j \in C_i} \log(UMI_j)}{\sum_{i \in T_6} \sum_{j \in C_i} \log(UMI_j)} \quad \textbf{S33}$$

Agreement Between Gene Expression and Optimal Flux Distributions

To quantify the quality of fitting between the integrated metabolic model and gene expression data, flux distributions obtained were assessed with regard to genes and reactions in G_{on}, R_{off}, and R_{low} sets used as inputs for integration (see above). For each tissue, evaluations were done based on fluxes only within the corresponding compartment (*i.e.*, *I* reactions for the intestine and *X* reactions for all other tissues). To evaluate the agreement of fluxes with the highly expressed genes, we first determined all active genes belonging to G_{on}. For each gene, absolute fluxes through reactions associated with the gene were summed. If this sum was greater than or equal to 1E-5, then the gene was considered active, as this value indicates a small but significant flux in the scale of our flux distributions and with respect to the solver parameters used (**Table S2**). The flux minimization at the end of every integration algorithm effectively reduces all non-

contributing fluxes to orders of magnitude less than this value. Genes in G_{on} that are not active indicate potential false negatives, *i.e.*, highly expressed genes which were not associated with any flux carrying reactions (**Figure 2D**). This analysis excludes highly expressed genes that are related to lowly or rarely expressed genes with an AND connection in the GPRs of all reactions they are associated with. Since reactions dependent on rarely or lowly expressed genes are assigned to R_{off} and R_{low} sets, such genes do not have any reactions left that are expected to carry flux and are therefore automatically dropped from integration (i.e., these genes are not part of G_{on} to begin with). The number of this type of genes was lower than 10% of highly expressed genes overall (**Figure S3B**). In addition, most of these genes ended up carrying flux in the OFD of iMAT++ (**Figure S3B**, results with iMAT was similar, data not shown). These fluxes indicate disagreements with R_{off} or R_{low} and were evaluated separately.

To address the fitting of flux distributions to rarely expressed reactions (R_{off}) we determined active reactions in the tissue compartment, again based on a flux threshold of 1E-5. Active reactions that were in the R_{off} set indicated potential false positives (**Figure 2D**). To evaluate the performance of integration with lowly expressed reactions (R_{low}), we first summed the absolute value of fluxes in these reactions (Σ_{low}). To compare this sum to the flux load of the integrated network, we also calculated the expected amount of flux in as many flux carrying reactions (Σ_{flux}). Given also the number of flux-carrying reactions in the tissue (N_{flux}) and of reactions in R_{low} (N_{low}), we calculated the depletion rate of flux in R_{low} based on **Equation S34** (**Figure 2D**). Although the iMAT algorithm does not distinguish between R_{off} and R_{low} (**Figure E4B**), we applied to this algorithm the same evaluation based on R_{off} and R_{low} sets used in iMAT++ for comparison purposes.

$$Depletion\ Rate = 1 - \frac{\Sigma_{low}}{\Sigma_{flux}\frac{N_{low}}{N_{flux}}} \qquad \textbf{S34}$$

Validation of Predicted Flux Distributions Based on Known Tissue Functions

We compared OFDs from iMAT++ to determine reactions with fluxes that were biased toward one or two tissues (*i.e.*, significantly higher or lower in one or two tissues than the other tissues) or nearly uniform across all tissues. First, such reactions were programmatically extracted from the entire set of reactions in iCEL1314. Briefly, for each reaction that had a non-zero flux in at least one tissue, the flux across all seven tissues (obtained from the pertaining compartment in the dual model, see above) was normalized by the maximum value (based on absolute flux values). This normalization yields a number between -1 and 1 for each tissue, wherein a negative value indicates flux in the backward direction for reversible reactions. Normalized fluxes across the seven tissues were then distributed to seven equal bins in this range. Reactions that yielded a maximum frequency greater than or equal to 5 in one of these bins were selected, as their fluxes were either biased toward one or two tissues or similar in all tissues. Second, these reactions were clustered based on the normalized flux values. *SciPy* library of Python programming language (version 2.7) was used for hierarchical clustering of this data with "single" linkage and "euclidean" distance. Finally, clusters in the resulting heatmap (**Figure 3A**, **Table EV6**) were manually analyzed with respect to tissue-relevant functions found in the literature.

Calculation of biomass yield

To calculate the yield of biomass for a tissue, we compared the biomass output of the dual-tissue model compartment pertaining to that tissue (*I* for intestine, *X* for other tissues) to the nutritional input of the same compartment. First, biomass production was calculated based on the flux of the BIO0010_X or BIO0010_I reaction depending on the tissue of interest (**Table EV5**). One unit of flux in this reaction represents the production of 1000 mg total dry biomass (Yilmaz & Walhout, 2016). Since the assumed biomass composition of *C. elegans* leaves a room of 5% to unknown and inorganic constituents (Yilmaz & Walhout, 2016), the flux of BIO0010 was multiplied by 950 to obtain the amount of biomass produced in mg. Second, the nutritional input was determined based on the sum of the fluxes of nutrients imported into the compartment. Nutrient flux was calculated by multiplying the flux of the import reaction that brings the nutrient into the compartment (*i.e.*, the flux of a transport or sink reaction in the relevant direction, in mmol/g dW/h) by the molecular weight of the nutrient (in mg/mmol). Water, oxygen, proton, potassium and sodium were not included in this analysis. The calculated amount of biomass produced was divided by the total nutrient flux to obtain the biomass yields (**Figure 3B**). Finally, we calculated the overall biomass yield (**Figure 3B**) by dividing total produced biomass by the nutritional input during the intestine integration, which represents overall requirements of the animal. To calculate total produced biomass, we added the weighted sum of biomass from the six non-intestinal tissues (*i.e.*, using tissue weights, **Figure E4A**) to intestine biomass.

Flux Variability Analysis of Tissue-Level Metabolic Network Models

The OFD of a tissue represents a parsimonious solution with minimum total flux according to **Equation S29**, which best agrees with categorized gene expression data. However, if we allow total flux in a tissue to change by a small value (Δ_{total}), we can obtain other flux distributions that agrees with highly, lowly, and rarely expressed genes as well as the original OFD. We consider such distributions as alternate optimal solutions (ALT) for that tissue. Exploration of ALT solution space is important when a reaction that seems to have no flux in an OFD can carry flux in ALT, or when the flux in a reversible reaction can be reversed in ALT. Obviously, the OFD alone may not be sufficient to explore all potential metabolic functions of a tissue. To determine the flux range of every reaction in the feasible solution space we performed FVA (Mahadevan *et al.*, 2002) on every tissue. We maintained all fitting constraints at the end of iMAT++ integration (**Equation S29**) and constrained total flux by the minimum obtained from **Equation S29** (Z^{min}_{total}) plus Δ_{total}, which was the same value used in **Equation S28** (i.e., Δ_{total} =0.05Z^{min}_{total}). As the objective function, we minimized and then maximized the flux of each reaction (*i*) resulting in the FBA problem shown in **Equation S35**. For each reaction, the flux range obtained this way (v_i^{min} to v_i^{max}) shows the flux values that reaction can take in ALT (**Table EV5**). As an exceptional point for the intestine, the constraints that defined the summed demands of other tissues (**Figure 2A**) were removed during FVA, as these demands may vary as well.

$$v_i^{min} = \min(v_i)\ ,\ v_i^{max} = \max(v_i) \quad s.t.$$
$$Z_{fit} = Z_{fit}^{max}, Z_{low} \le Z_{low}^{min} + \delta_{low}\ , Z_{latent} = Z_{latent}^{max}\ , Z_{total} \le Z_{total}^{min} + \Delta_{total} \quad \textbf{S35}$$

Next, for each tissue, we used OFD and FVA results together to categorize each reaction as carrying flux in OFD, carrying flux in ALT, or not carrying flux in the feasible solution space (SLNS) (**Figure 3C**, **Tables EV7** and **EV8)**. To be able to evaluate

reversible reactions separately in either direction, we converted every reaction to a forward and a reverse reaction, or whichever is applicable, with reactants and products swapped for the reverse reaction. To categorize a forward or reverse reaction we used three values: the flux of the reaction in OFD (v_{OFD}, equal to the flux in OFD for forward and to the negative of this flux for reverse reactions), the minimum flux it can take (v_{min}, equal to v_i^{min} from FVA for forward and to $-v_i^{max}$ for reverse reactions), and v_{max} (equal to v_i^{max} for forward and to $-v_i^{min}$ for reverse reactions). We also used three flux thresholds: one below which flux is considered insignificant (*tol_zero* = 1E-7), one above which flux is considered significant (*tol_flux* = 1E-5), and the regular reaction-specific flux threshold from integration (ε-values, see above). For a given reaction, the algorithm for reaction categorization was briefly as follows:

> If v_{OFD} > *tol_flux*, then the reaction carries flux in OFD.
>
> Else if v_{OFD} > *tol_zero* and v_{min} > *tol_zero*, then the reaction carries flux in OFD.
>
> Else if v_{max} ≥ max(ε-*tol_flux*, *tol_flux*), where the *max()* function indicates the greater of the two values in parentheses, then the reaction carries flux in ALT. Here, the regular flux threshold for the reaction (ε) was reduced by a tolerance (*tol_flux*) so that a reaction that can carry a flux close to ε would be categorized as carrying flux in ALT. The use of the maximum function means that values lower than *tol_flux* were not allowed.
>
> Else (meaning, v_{max} < max(ε-*tol_flux*, *tol_flux*)), the reaction does not carry flux in SLNS.

Using the results of this algorithm, we defined conservative tissue networks with reactions in OFD and ALT (**Figure 3C**, see below). Intestine reactions were evaluated for both intestine solution space and for the solution space of the other tissue integrations. If an intestine reaction carried flux in ALT for intestine, but not in SLNS for another tissue, then this reaction was considered as not carrying flux in SLNS.

Flux Potential Analysis for Quantitative Integration of Expression Data

We developed and used flux potential analysis (FPA) to determine the relative flux potential of every reaction in every tissue, given expression levels of metabolic genes (**Figure 5A**). In principle, the same approach can be applied to any gene expression dataset with different tissues or conditions, and with any metabolic network model, although specific flux constraints that apply to our dual-tissue model (**Figure 2A** and below) would need to be removed or adjusted. We provide tools for the general applicability of FPA as well as for use with the *C. elegans* dual tissue model (see Code and Model Availability section below).

Before performing FPA, every reversible reaction in the metabolic network model is divided into two reactions, each representing one of the two directions (forward or reverse). The one representing the reverse direction has reactants and products of the original reaction in the reverse order. Thus, all reactions in the converted model have only a forward direction, which conveniently creates a solution space without negative flux values. As an additional, *C. elegans*-specific model modification, we removed ATP usage from bacterial digestion (*i.e.*, converted the coefficients of atp, adp, h2o, h, and pi in reaction

DGR007_I in **Table EV5** to zero) to prevent digestion-related ATP synthesis from interfering with FPA.

After necessary modifications, FPA was applied to every reaction of the modified model one-by-one, for each tissue, in the dual-tissue model context. For intestine calculations, only *I* and *L* (transport only) reactions were evaluated. For other tissues, only *X* reactions were evaluated. To calculate the flux potential (*FP*) of a queried (target) reaction (*k*) in a particular tissue, FBA is performed using the maximization of the flux of this reaction (v_k) as the objective function (**Figure 5A**). This maximization is achieved under a set of constraints in various equations (**Equation S36**). Regular mass balance constraints (**Equation S5**) apply. Regular reaction boundary constraints (**Equation S6**) simplify to **Equation S37** as all reactions in the converted model are irreversible. Notably, this constraint applies also to the non-growth associated maintenance ATP reaction (RCC0005), the lower boundary of which is normally a positive value that represents constant ATP requirements of the tissues. This change, together with the abovementioned elimination of the digestion-related ATP requirement, prevents the interference of background ATP synthesis with FPA. Thus, the ATP production potential of a tissue is relevant only when the flux of the target reaction creates an ATP demand. The upper boundary of flux in **Equation S37** (v^{UB}) needs to be set at a large number for most reactions, to allow any reasonable flux value. As in other applications in this study, we used 1000 flux units for this upper boundary. However, a subset of reactions can be selectively blocked by using a v^{UB} of 0. In particular, it is necessary to block the reverse of the target reaction, if it exists, so that the forward and reverse forms of the same original reaction do not form a flux loop. As an important set of constraints in this study, we blocked all reactions that are not in OFD or ALT solutions in the relevant tissues (**Figure 3C**, **Tables EV7** and **EV8**), so that reactions that are likely not part of the relevant tissue metabolic networks cannot contribute to flux potential. In addition, when the target reaction is an intestine reaction, all reactions in the *X* compartment are blocked. Further boundary constraints were implemented for metabolite-level analyses as will be described below.

$$FP_k = v_k^{max} = \max(v_k) \quad s.t. \\ Eqns\ \text{S5, S37, S38} \qquad \textbf{S36}$$

$$0 \le v_i \le v_i^{UB}; i \in R_{all} \qquad \textbf{S37}$$

The constraint that actually controls the tissue-level differences in the flux potential of the target reaction is given in **Equation S38**, which shows that a weighted sum of all fluxes in the metabolic network is limited by a constant (a), which we refer to as “flux allowance”. We arbitrarily used a flux allowance of 1, which resulted in large enough flux values that could be numerically differentiated from zero. The weight of a reaction in **Equation S37** (w_i) is related to the coefficient of gene expression (c_i) and the distance of the reaction to the target reaction (d_{ik}), in addition to the distance order (n) (**Equation S39**). Next, we describe gene expression coefficients and reaction distances, before showing how relative flux potential is calculated.

$$\sum_{i \in R_{all}} w_i v_i \le a \qquad \textbf{S38}$$

$$w_i = \frac{c_i}{(1 + d_{ik})^n} \qquad \textbf{S39}$$

Gene expression coefficients

Based on **Equation S38**, a larger weight on a particular reaction indicates that a bigger portion of the flux allowance will be spent per flux carried by that reaction. Since allowance is limited, weights in **Equation S38** act as penalties on placing flux in reactions. The idea of using the gene expression coefficient in the reaction weight (**Equation S39**) is about penalizing the reaction more when the gene encoding the enzyme that carries out the reaction is less expressed and *vice versa*. Therefore, a tissue (or condition) that consistently has relatively high expression levels for genes associated with reactions that contribute to the flux potential (*i.e.*, the maximized flux in target reaction, **Equation S36**) would use the flux allowance more efficiently and achieve a larger flux potential.

To penalize low gene expression, we define the *c*-value of each reaction using **Equation S40**, where *E* is the total expression of the genes encoding the enzyme associated with the reaction, τ is the tissue that accommodates the reaction, and *max* indicates the maximum value for all tissues. In the case of a GPR association with only one gene, *E* simplifies to the expression of that gene (then **Equation 40** is equivalent to 1/normalized gene expression, which is used in **Figure 5A**). If GPR consists of multiple paralogs or isozymes connected with OR (*e.g.*, A I B, with A and B denoting hypothetical genes), then **Equation S41** is used to calculate *E* with *x* denoting the expression of a particular gene. If the GPR connects multiple genes that encode different proteins with AND (*e.g.*, A & B), as in the case of subunits of an enzyme, or multiple enzymes acting in series to carry out a lumped reaction, then the maximum coefficient (penalty) is used, as shown in **Equation 42**. In rare but complicated GPR associations in the form of (A & B) | C, the minimum expression of A and B is summed with the expression of C to obtain *E*, and then **Equation S40** is used to calculate the coefficient. The GPR parsing algorithm we provide with the FPA tools automatically implements these rules by first dividing the GPR association into non-redundant blocks (separated by AND), then calculating the coefficient for each block, and finally taking the maximum according to **Equation S42**. This algorithm treats the exemplified GPR association, (A & B) | C, as a whole block. We used the provided TPM values (Cao *et al.*, 2017) for gene expression (*x*, **Equation S41**), but matching metrics (*e.g.*, FPKM) can be used with other datasets.

$$c_i^\tau = \frac{E_i^{max}}{E_i^\tau} \qquad \textbf{S40}$$

$$E_i^\tau = \sum_{j \in Isozyme} x_{ij}^\tau \qquad \textbf{S41}$$

$$c_i^\tau = \max_{q \in Protein} \left(\frac{E_{iq}^{max}}{E_{iq}^\tau}\right) \qquad \textbf{S42}$$

<u>Exceptional gene expression coefficients</u>

Reactions with no gene associations were given a gene expression coefficient of 1, with few exceptions. Exchange reactions (excluding side metabolite uptakes) were given a coefficient of 0 (*i.e.*, not penalized). Thus, metabolite exchange was penalized only based on transport reactions. Bacterial ingestion and degradation reactions were also given a coefficient of 0 so that nutritional input is penalized only for the uptake of side nutrients. When calculating flux potential for a reaction in the *X* compartment, intestinal transport reactions were not penalized to avoid excessive penalization of a non-intestinal tissue for material transport. Thus, every tissue is penalized only once for the intake of a metabolite: at the transport reaction that imports the metabolite into the tissue.

Metabolic distance

According to **Equation S39**, the reaction weight in the flux sum of **Equation S38** decays with the order *n* as the weighted reaction gets further away from the target reaction. This decay is the same for all tissues (or conditions) analyzed. As a result, distant reactions spend smaller portions of the flux allowance (unless *n* is 0), which means gene expression differences in these reactions are less significant in the calculation of flux potential than differences in reactions proximal to the target reaction. The idea behind this formulation is that the flux of the target reaction is likely more affected by metabolites and enzymes that are nearby in the metabolic network than those further away. For instance, when estimating the flux potential of a reaction in the valine degradation pathway (**Figure 2C**), one would not expect TCA cycle flux to be informative necessarily, although end products of valine degradation, such as succinyl-coa, are eventually drained by this central pathway. Because of the distance decay, the role of differential gene expression in the TCA-cycle is reduced and that of proximal reactions within the valine degradation pathway is increased during the calculation of flux potential. This approach is reasonable since TCA-cycle flux may be regulated by many other cellular processes and needs than valine degradation alone. Thus, if one tissue has higher expression of TCA-cycle genes and the other has higher expression of valine degradation pathway genes, we expect the latter to have a larger flux potential in valine degradation, consistent with the usage of distance metric in FPA.

We define the metabolic distance between two reactions as the shortest path between them. Finding shortest paths in metabolic networks is not a trivial problem, especially because of reaction reversibility and the existence of multiple substrates and products in many reactions (Frainay & Jourdan, 2017). We developed an algorithm (see Code and Model Availability section) that initially converts each reaction in the modified model (*i.e.*, the model with each reversible reaction converted to two irreversible reactions) to a node that is directionally linked to other reactions (nodes) that produce its reactants ("from" nodes) and consume its products ("to" nodes). When establishing this reaction network, we ignored 21 metabolites that are frequently used and can potentially connect otherwise unrelated reactions, in order to avoid irrelevant paths with short cuts (Frainay & Jourdan, 2017). In the nomenclature of BIGG models (King *et al*, 2016), which is also used in our metabolic model, these metabolites are adp, amp, atp, co2, coa, crn, etfox, etfrd, fad, fadh2, gdp, gtp, h, h2o, nad, nadh, nadp, nadph, o2, pi, and ppi (**Table EV3**). Once the reaction network is formed, our algorithm allows the user to traverse this network from a particular reaction node (*i*) to all other nodes (*j*) that can be reached (Traversing excludes the reverse of the starting reaction, if this reaction comes from an originally reversible reaction). Since the path to a node is fixed once it is reached the first time, the path from *i* to any node *j* is the shortest possible in the number of reactions (steps). Therefore, this number indicates the distance from reaction *i* to reaction *j*. We found the distance from every reaction to every other by traversing the network from all reactions by changing the starting node *i*. If a reaction *j* is not reachable from *i*, then the distance from *i* to *j* can be

taken as a prescribed maximum, which was 31 in this study (based on the maximum actual distance found within the network). Because of reaction reversibility rules, the length of the shortest path from reaction *i* to *j* may not be the same as that from *j* to *i*. Therefore, we took the minimum of these two distances to define the final distance between *i* and *j*.

Importantly, the network traversing algorithm sometimes uses a path that crosses a reversible reaction of the unmodified model twice (*i.e.*, uses each one of the forward and reverse reactions that were derived from a reversible reaction in the original model). Since these paths are not real, the distance is miscalculated in such cases. It is not computationally feasible to avoid this issue by an exhaustive search of alternative paths, as this is an NP-complete problem (Frainay & Jourdan, 2017). Our algorithm handles this problem by finding all paths with loops over originally reversible reactions, and then eliminating the first reaction in the path that is part of a loop. Since the path found after reaction elimination can still have loops, this procedure is iteratively repeated until a valid path is found. If only one reaction is eliminated and the final destination (node *j*) is the reverse of the eliminated reaction, then the shortest path is still guaranteed. Otherwise it cannot be guaranteed. We know that less than 1% of shortest paths found were affected by this problem. Manual analysis of hundreds of potentially problematic distances in the outcome showed that all were correct. Therefore, we think this problem turned out to have a negligible effect in our analyses, if any.

<u>Exceptions to distance rules</u>

Distance decay of flux penalties was not implemented for a subset of reactions during FPA. When the target reaction was in *X* compartment, distances to all reactions in the *I* compartment were made 0, in order to avoid the excessive usage of intestinal network when calculating flux potentials for other tissues. Also, to discourage the uptake of side nutrients, distances to all uptake reactions were converted to 0 in all calculations.

Calculation of relative flux potentials

The flux potential of the target reaction (FP_k) is the maximum flux that can be obtained in this reaction in a tissue using proper constraints (**Equation S36**). In addition to being calculated for all tissues, FP_k is also calculated for a super system, which is a hypothetical tissue (or condition) with a gene expression coefficient of 1 (*i.e.*, the lowest possible flux penalty from gene expression; **Equation S39**) for all metabolic reactions associated with genes. Once FP_k is found in all tissues and the super system, the relative flux potential of the target reaction in a tissue ($rFP_{k\tau}$) is calculated by dividing the flux potential in the tissue (τ) by that of the super system (*S*) (**Equation S43**). Since by definition no tissue can have a higher flux potential than the super system, relative flux potential is a value between 0 and 1.

$$rFP_{k\tau} = \frac{v_{k\tau}^{max}}{v_{kS}^{max}} \qquad \textbf{S43}$$

Metabolite-level FPA

FPA of reactions that drain or introduce metabolites allow metabolite-level evaluations. The flux potential of an export (transport from *I* or *X* to *E* in the dual-tissue model) or a demand reaction indicates the production potential for the metabolite drained. Likewise,

the flux potential of an import (transport from *E* or *L* to *I* or *X*) or a sink reaction indicates the degradation potential for the metabolite introduced. FPA is applied to these reactions with specific constraints that prevent short cuts which could otherwise impair the analysis. For example, if trehalose import is the target reaction, then the trehalose demand reaction should be blocked, as the latter can drain imported trehalose without any metabolic processing, thereby causing an overestimation of trehalose degradation potential. When trehalose demand is blocked, the degradation of the imported trehalose needs to take place using enzymatic reactions, thus reflecting the expression levels of the relevant enzymes on the trehalose degradation potential being calculated. Therefore, when calculating the *FP* of a transport, sink or demand reaction, we blocked all other reactions of this type in the same tissue compartment.

Our algorithm also allows metabolite-level analysis when the metabolite of interest is not associated with any transport, demand or sink reactions. In that case, a demand or sink reaction of the metabolite can be incorporated into the model for simulating the production or degradation of the metabolite, respectively. The distances of the newly introduced reaction to others in the network are calculated based on the available distance matrix. For a demand reaction, the distance of a given reaction is calculated as 1 plus the minimum of the distances from all reactions where the metabolite of interest is a reactant. For a sink reaction, the same calculation is done except that the minimization is over reactions where the metabolite is a product. In this study, we analyzed production potentials for 26 biomass precursor metabolites (**Table EV8**, reactions with metabolite names as IDs) using additional demand reactions.

Systematic Analysis of Tissue Function Based on Flux Potentials

We carried out comprehensive FPA for all reactions and tissues, using a distance order of 1.5 (*i.e.*, $n = 1.5$, **Equation S39**). As explained in the main text, this choice of order was primarily based on the metabolite-level analysis with collagen that shows relative collagen production potentials as a function of increasing n (**Figure 5D**). In addition, *rFP* curves with a transitional behavior seem to change direction around n = 1-1.5 (**Figures 5B-5D**), which we interpret as an indication that this region corresponds to a transition from a global integration (where distance penalties are very low as the distance order is low, and therefore, all reactions equally matter) to a local integration (where distance penalties are very high because of high order, and only nearby reactions matter). Thus, we chose a semi-local integration region where reactions farther from the target still mattered, albeit with a reduced impact.

After collecting all *rFP*-values, we performed a simple variation analysis to capture reactions that have significantly higher flux potential in one or two tissues than others. We used an algorithm that considered only reactions for which the difference between the maximum and minimum *rFP*-values in all tissues was at least 0.35. For these reactions, tissues were sorted according to descending *rFP*-values. The first tissue was labeled as the primary site for the reaction, if its *rFP*-value was at least 40% or 0.2 units greater than the median *rFP*-value, and greater than the *rFP*-value of the third tissue by at least 0.1. The second tissue was labeled as the secondary site, if its *rFP*-value was at least 20% or 0.1 units higher than the median *rFP*-value, and at least 0.07 higher than the *rFP* -value of the third tissue. Primary and secondary tissues, if available, are reported for each applicable reaction in **Tables EV7** and **EV8**. These rules were trained by collagen production data, where the primary and secondary sites were assumed to be hypodermis and glia, respectively, based on **Figure S4A**, and GABA (4abut) production data, where the primary site was assumed to be neurons, as GABA is a neurotransmitter. Other predictions of primary and secondary sites were automatically found.

We also compared primary and secondary tissue labels with tissue-level OFDs to derive cases where FPA and iMAT++ results are in at least qualitative agreement. Specifically, for a regular reaction (**Tables EV7**), we starred (*) the primary or secondary tissue, if the reaction also carries flux in the OFD of that tissue. For a metabolite-level reaction (**Tables EV8**), we only required that the reverse reaction, if it exists, does not carry flux in the tissue OFD. Thus, starred primary or secondary sites are tissues where the calculated high flux potential is consistent with the network-level integration. We have the highest level of confidence in these predictions.

Summary of Findings with MERGE

Results from tissue OFDs, FVA, and FPA are summarized in **Tables EV7 and EV8**. We inserted in these tables the results of our primary and secondary tissue analysis described above. This analysis combines results in a particular way, with emphasis on reactions and metabolites biased toward one or two tissues. However, the data in these summary tables can be interpreted using other approaches with different stringency levels (*e.g.*, not requiring that reaction-level *rFP* results and OFD fluxes are consistent) or just by manual analysis with the help of different table fields provided. Results presented in **Figure 7** are all based on conservative predictions from starred primary and secondary tissues, which were verified using the literature, as indicated in the main text.

Robustness and Usability of MERGE

We carried out sensitivity analyses to test the robustness of our main findings and to evaluate the importance of selected parameters or modules in our computational pipeline. In each analysis, we recalculated the results with a parameter or module perturbed, and asked how much the predictions of tissue-specificific metabolic functions would change. We first compiled a list of reactions and metabolites that represent the main conclusions summarized in **Figure 7** and then mapped resuls from each sensitivity analysis to this list (**Figure EV5A**). The listed metabolic functions were assigned to a primary or secondary tissue with high confidence, meaning, one or two tissues are forefront with regard to *rFP* and OFD predictions are not in contradiction with the evaluated function. We also looked at the overall difference in all high confidence predictions (**Figure EV5B**) and at the difference of rFP values from all reactions (**Figure EV5C**). In **Figure EV5B** and **Figure EV5C,** *SciPy* library of Python programming language (version 2.7) was used for hierarchical clustering of the data with “jaccard” and “cosine” distance metric, respectively.

Flux thresholds

The integration algorithms (iMAT and iMAT++) used in this study need the definition of a minimum flux value (ε) to determine if a reaction is carrying flux (see above, Integration Algorithm/Determination of flux thresholds). The flux thresholds over the network were determined based on two parameters: the default ε value, which applies to most reactions, and a divisor that is used to divide the maximum flux capacity of a reaction given a bacterial intake limit of 1 unit. For each reaction, the smaller of the divided flux capacity and the default value is used as the flux threshold. Our results were obtained with a default ε value of 0.01 and a flux capacity divisor of 2. Changing the default ε to 0.02 or the divisor to 1 or 3, did not have a significant impact on the main results (**Figure EV5A**) or on the overall (**Figures EV5B** and **EV5C**). Increasing the divisor beyond 3 causes numerical problems since some flux thresholds approach zero. Increasing default ε further throws the flux distribution scale off. For example, the overall bacterial intake rate assumed during the flux capacity calculations is sufficient to support a decreasing number

of reactions with larger ε values. Thus, a proper test for bigger thresholds can only be done by increasing the allowed bacterial uptake rate proportionally. When this adjustment is done, a 5 times increase in default ε has negligible effect on the results (**Figure EV5**). In conclusion, MERGE is robust to proper flux thresholding.

Network building algorithms

The most time demanding step of MERGE is the application of FVA, to every reaction of the model, to build tissue networks. Our preliminary analyses with human model Recon 2.2 (Swainston *et al*, 2016) suggest that this step may be unfeasible with the default parameters and algorithm when applied to more complex models. Interestingly, omission of the FVA step and the direct application of FPA on the entire network had a modest impact on our major findings in this study (**Figure EV5A**). However, it is not ideal to ignore network differences since some predictions are obviously affected and the differences are relatively large on the overall (**Figures EV5B** and **EV5C**). We therefore developed two other alternatives for the FVA step with which MERGE provides very similar results to those from the original algorithm in a time-efficient manner. We refer to the default algorithm as Speed Level 1 and the two alternatives as Speed Level 2 and Speed Level 3, with the computational speed increasing at each level. We validated the applicability of these changes with *C. elegans* (**Figure EV5**, see below) and we provide a demonstration of the application of MERGE with speed level 2 on the human model Recon 2.2, in our GitHub repository (see below).

Speed level 2: In this level, we perform iMAT++ with the original algorithm except for relaxing a solver parameter, MIPGap (**Table S2**), from 1E-12 to 1E-3 during the minimization of latent reactions. We recommend this parameter change for all applications with large and complex models such as the human model, as integration occasionally fails to finish with some gene expression sets when the default parameter is used. In addition to this parameter adjustment, FVA in this level is applied at the PFD stage instead of OFD. Thus, the latent reaction corrections is skipped during FVA. In principle, this is not a significant compromise since FVA readily captures the flux carrying capacity of latent reactions and forcing latent reactions to carry flux should not significantly affect the flux carrying capacity of others qualitatively. Indeed, the modified algorithm reproduced all major results of this study (**Figure EV5A**) with minor differences overall (**Figures EV5B** and **EV5C**) comparable to those created by numerical differences created by changing arbitrary flux thresholds (see above). MERGE at speed level 2 is applicable to complicated models like Recon 2.2 to get results for >10 tissues in a reasonable time frame (several hours) when a computer server with multiple cores is available.

Speed level 3: The difference of this level from the previous one is a modification of iMAT++ algorithm regarding the constraints on low-flux reactions (*i.e.*, after **Equation S24**, as a replacement for $Z_{low} \leq Z_{low,min} + \delta_{low}$). Rather than just limiting the sum of flux in these reactions, harder constraints are applied as shown in **Equations S44** and **S45**. Together with the modifications in speed level 2, this adjustment improves the computational speed by orders of magnitude compared to the original algorithm. We found that Speed Level 3 also recovered all main results of this study (**Figure EV5A**), and the differences overall indicated a reasonable deviation from the original results, more than Speed Level 2 but much less than using the full network with every tissue (**Figures EV5B** and **EV5C**). Thus, we recommend MERGE at speed level 3 when basic computational equipment is used.

$$v_i = 0;\ i \in R_{low}\ and\ v_i^{Eq.\ S24} = 0 \qquad \textbf{S44}$$

$$|v_i| \leq |v_i^{Eq.\ S24}| + \delta_{tol};\ i \in R_{low}\ and\ v_i^{Eq.\ S24} \neq 0 \qquad \textbf{S45}$$
$$\delta_{tol} = 10^{-8}$$

Distance order

The distance order is an important parameter in FPA as it causes reaction weights to decay with distance by power law (**Equations S38** and **S39**). This parameter was therefore carefully calibrated using the desired prediction of hypodermis as a primary site for collagen production, and a value of 1.5 was assumed (**Figure 5D**). Using a distance order of 1 or 2 did not affect the vast majority of the results, but four primary or secondary site predictions were completely lost in each test (**Figure EV5A**). The overall difference from the original results was about 15-20% based on high confidence predictions (**Figure EV5B**). Taking the distance order to extremes at 0 and 10 made bigger impact. However, most results were reproduced with a distance order of 0, indicating that omitting metabolic distance from FPA calculations altogether still yields useful predictions. At a distance order of 10, most predictions were affected mainly because of losing metabolite-level resolution (**Figures 5C** and **5D**). Thus, MERGE results with *C. elegans* were generally stable at distance orders less than 2, but a careful tuning of this parameter is needed for optimal predictive power.

Relative expression analysis

We tested how much impact the recategorization of some genes based on relative expression profiles had on the final results. Ommitting the relative expression analysis altogether had a modest impact on our key findings, with one prediction losing tissue enrichment and two other being demoted to low confidence (**Figure EV5A**). However, a significant percentage of overall findings at high confidence level were changed (**Figure EV5B**). Possible negative impact of incomplete gene categorizetion is partially buffered by FPA, as the relative values of expression are readily used by this method. The impact is on the OFD and tissue networks, which are based on gene categories, and which limit the FPA solution space and affect the confidences in the final predictions. Thus, although our verifiable findings were not significantly affected by the omission of relative expression analysis, the additional information provided by this method had significant impact on the results overall.

Code and model availability

We provide four computational tools that implement the major algorithms developed in this study (**Table S3**). These tools and related input data are deposited in GitHub (https://github.com/WalhoutLab/MERGE). Data presented in this study were primarily generated with Python language using the original versions of programs in **Table S3** as well as other codes that are available upon request. iMAT++ and FPA algorithms were translated to MATLAB language and linked with the COBRA toolbox commonly used in metabolic network modeling (Heirendt *et al*, 2019) for user convenience. The differences between the results of the original Python library and the presented programs are only minor. In most linear optimizations, Gurobi solver version 8 (https://www.gurobi.com/) was used, with the key parameters set as indicated in **Table S2**.

Metabolic models with updates (iCEL1314 and the dual-tissue model version of this model) are available in **Tables EV2**, **EV3**, and **EV5**, as well as in WormFlux (http://wormflux.umassmed.edu/), which allows interactive search through the network

(Yilmaz & Walhout, 2016) . From this website, the models can be downloaded in .xlsx, .mat, and sbml versions. The .mat versions are also available in GitHub together with the programs provided.

The GitHub repository also includes a demonstration of the applicability of MERGE to human metabolic network. A walk through script is provided that achieves a preliminary integration of a human metabolic model (Swainston *et al.*, 2016) with a tissue-level transcriptomic dataset (Uhlen *et al*, 2015).

SUPPLEMENTARY FIGURES

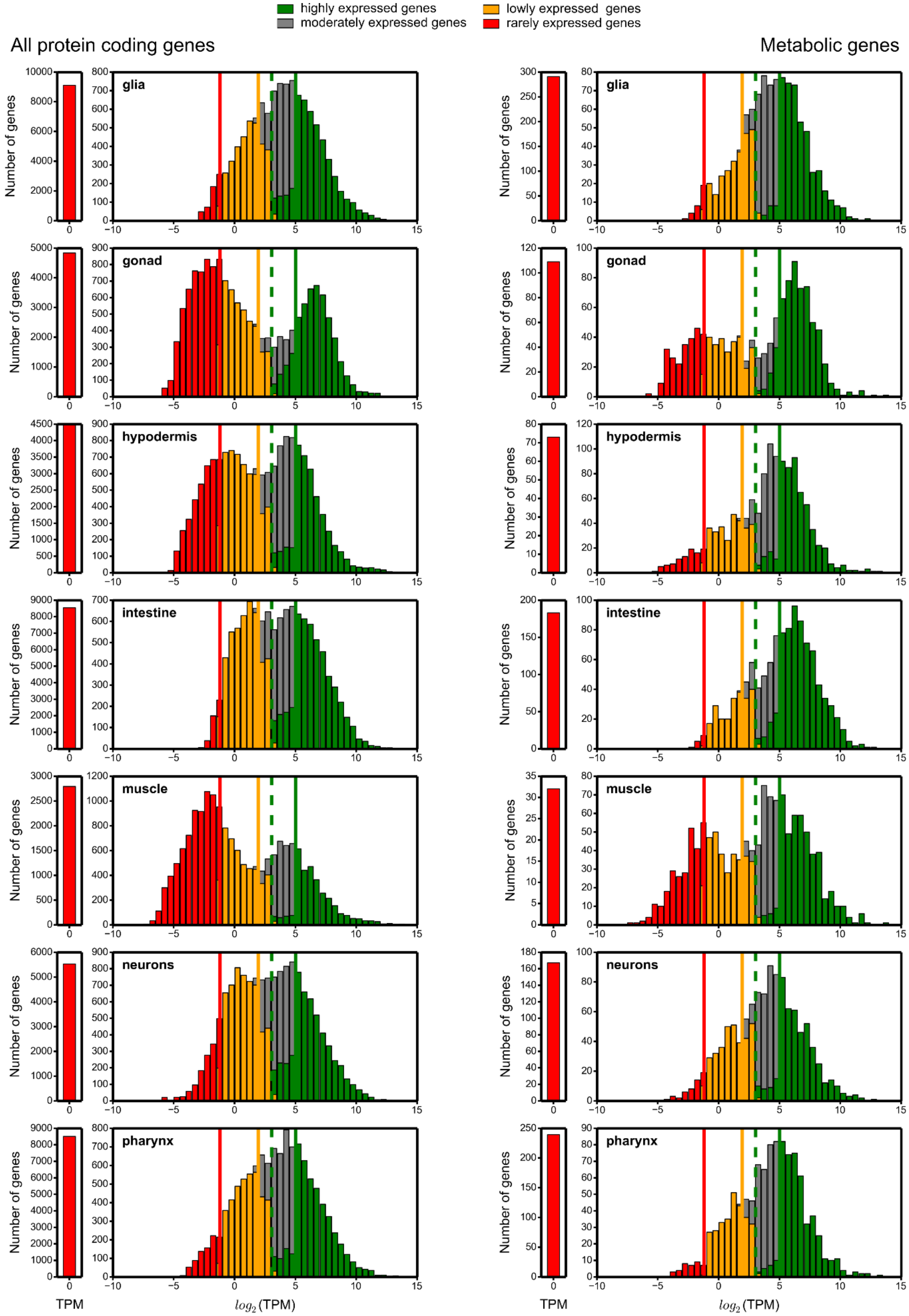

highly expressed genes
moderately expressed genes
lowly expressed genes
rarely expressed genes
All protein coding genes
Metabolic genes
glia
gonad
hypodermis
intestine
muscle
neurons
pharynx
Number of genes
TPM
log2 (TPM)

Figure S1

Labeled gene expression histograms for each of the seven tissues with all protein coding genes (left panel) and genes in iCEL1314 (right panel). See **Figure EV2** for detailed descriptions.

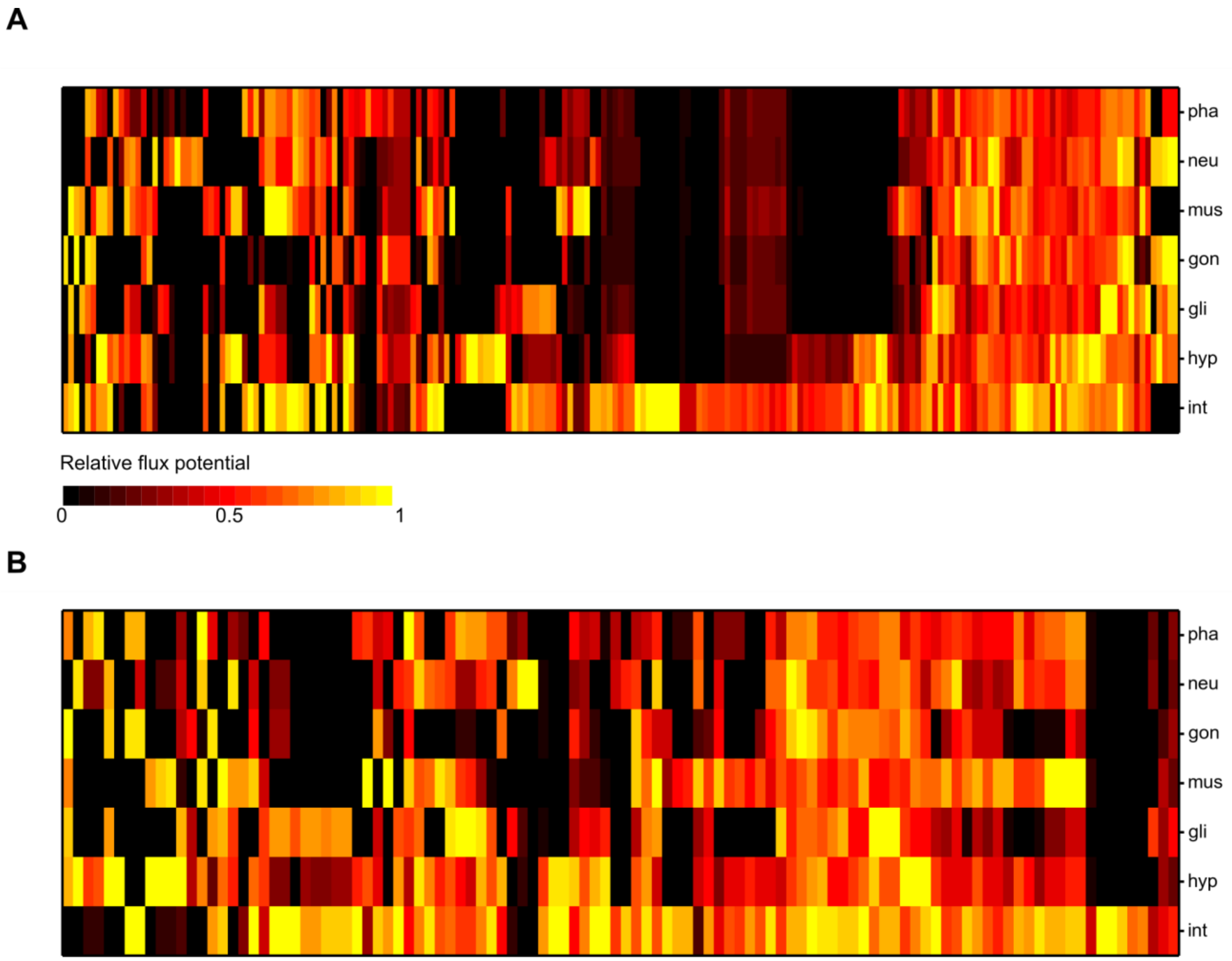

Figure S2

Relative production **(A)** and degradation **(B)** potentials of metabolites. Production potential of a metabolite (column) is based on the flux potential of export and demand reactions in the metabolic network model that consume the metabolite. Additional demand reactions that were introduced to drain biomass precursors are included. Degradation potential is based on the flux potential of import and sink reactions that bring in metabolites to the network. Only metabolites with high variation of relative flux potential are shown (see Supplementary text and **Table EV8**), totaling 200 metabolites in production **(A)** and 112 in degradation **(B)** heatmaps.

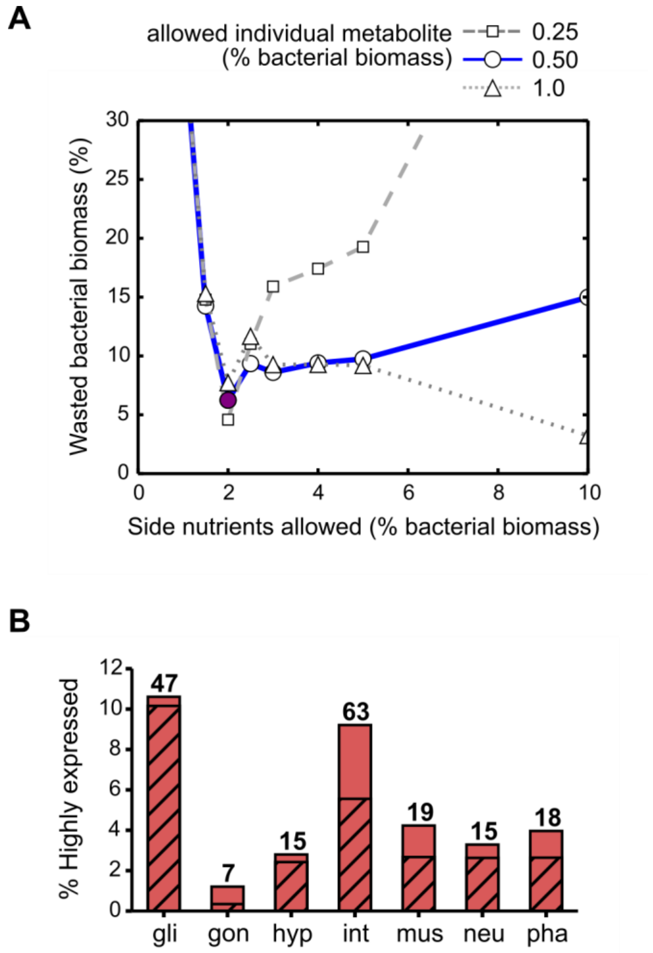

Figure S3

(A) Optimization of the relative uptake rate of side nutrients. Relative uptake rate is defined as grams of total side nutrient taken up per gram bacterial biomass consumed. Given the maximum relative uptake values allowed (x-axis) the dual model was integrated with data and the wasted bacterial nutrients (*i.e.*, bacterial components excreted from lumen to environment, (**Figure 2A**) was calculated as weight percentage of all bacterial biomass (y-axis) during the final integration with intestine data, which defines the overall metabolic activity (**Figure 2A**). Maximum allowed relative uptake rate of individual side metabolites was also varied as shown with the different lines. To maximize the dominance of bacterial nutrients in the dietary input, relative uptake rates of 2% for total side nutrients and 0.5% for individual side nutrients were selected for this study (purple circle). Although the use of 0.25% limit for individual side metabolites allowed lowering the waste further, this option was rejected due to the instability of the 0.25% curve at the waste minimum. **(B)** Highly expressed genes that were ignored during integrations as they are associated only with lowly or rarely expressed reactions. Lowly or rarely expressed genes in these reactions are connected to highly expressed genes with AND connection (*e.g.*, when multiple genes encode proteins of an enzyme complex), thereby restraining the assumed flux state. Number of genes represented by each bar is indicated at the top. Hatched section shows genes that ended up carrying flux after integration.

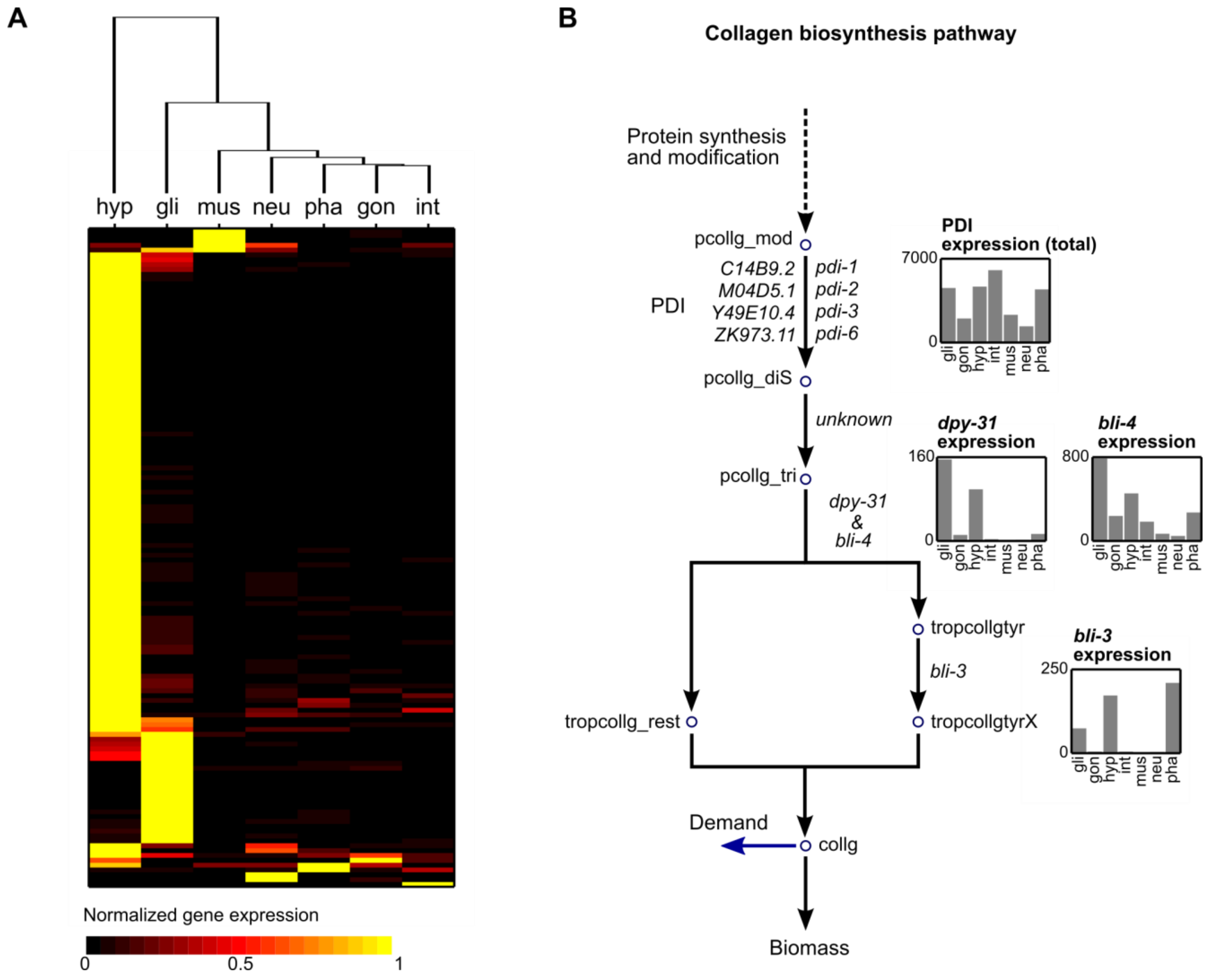

Figure S4

(A) Tissue-level expression profiles of 187 genes annotated as collagen coding (Holdorf *et al*, 2019) and highly expressed in at least one tissue. Each row represents a collagen. Columns are TPM values in tissues divided by the maximum in the row. **(B)** Collagen biosynthesis pathway in the metabolic network model. Blue arrow indicates the demand reaction added to uncouple collagen production from biomass production.

SUPPLEMENTARY TABLES

Table S1

Compartmentalization of metabolites, reactions, and genes in the dual-tissue model.

**Compartment**	**Metabolites**[a]	**Reactions**	**Genes**
Lumen (L)	252	506	0
Intestine (I)	1149	1845	1314
Other tissue (X)	1136	1832	1309
Extracellular space (E)	326	323	0

[a] Not unique (a metabolite that appears in two subcellular compartments is counted two times).

Table S2

Solver parameters used.

Parameter	Value	Description
FeasibilityTol	1E-9	Primal feasibility tolerance
OptimalityTol	1E-9	Dual feasibility tolerance
MIPGap	1E-12	Relative mixed integer programming optimality gap
Presolve	-1	Controls the presolve level

Table S3

Available codes for the algorithms developed.

Algorithm	Program name	Environment
Gene categorization	CatExp.py	Python 2.7
iMAT++	iMATplusplus.m	MATLAB, COBRA
FPA	FPA.m	MATLAB, COBRA
Distance calculation	MetabolicDistance.py	Python 2.7

SUPPLEMENTARY REFERENCES

Artyukhin AB, Zhang YK, Akagi AE, Panda O, Sternberg PW, Schroeder FC (2018) Metabolomic "Dark Matter" Dependent on Peroxisomal beta-Oxidation in *Caenorhabditis elegans*. *J Am Chem Soc* 140: 2841-2852

Brunk E, Sahoo S, Zielinski DC, Altunkaya A, Drager A, Mih N, Gatto F, Nilsson A, Preciat Gonzalez GA, Aurich MK *et al* (2018) Recon3D enables a three-dimensional view of gene variation in human metabolism. *Nat Biotechnol* 36: 272-281

Cao J, Packer JS, Ramani V, Cusanovich DA, Huynh C, Daza R, Qiu X, Lee C, Furlan SN, Steemers FJ *et al* (2017) Comprehensive single-cell transcriptional profiling of a multicellular organism. *Science* 357: 661-667

Frainay C, Jourdan F (2017) Computational methods to identify metabolic sub-networks based on metabolomic profiles. *Brief Bioinform* 18: 43-56

Gebauer J, Gentsch C, Mansfeld J, Schmeisser K, Waschina S, Brandes S, Klimmasch L, Zamboni N, Zarse K, Schuster S *et al* (2016) A genome-scale database and reconstruction of *Caenorhabditis elegans* metabolism. *Cell Syst* 2: 312-322

Harris TW, Baran J, Bieri T, Cabunoc A, Chan J, Chen WJ, Davis P, Done J, Grove C, Howe K *et al* (2013) WormBase 2014: new views of curated biology. *Nucleic acids research*

Heirendt L, Arreckx S, Pfau T, Mendoza SN, Richelle A, Heinken A, Haraldsdottir HS, Wachowiak J, Keating SM, Vlasov V *et al* (2019) Creation and analysis of biochemical constraint-based models using the COBRA Toolbox v.3.0. *Nat Protoc* 14: 639-702

Holdorf AD, Higgins DP, Hart AC, Boag PR, Pazour GJ, Walhout AJM, Walker AK (2019) WormCat: An Online Tool for Annotation and Visualization of *Caenorhabditis elegans* Genome-Scale Data. *Genetics*

Jensen PA, Lutz KA, Papin JA (2011) TIGER: Toolbox for integrating genome-scale metabolic models, expression data, and transcriptional regulatory networks. *BMC Syst Biol* 5: 147

Kanehisa M, Sato Y, Kawashima M, Furumichi M, Tanabe M (2015) KEGG as a reference resource for gene and protein annotation. *Nucleic Acids Res*

King ZA, Lu J, Drager A, Miller P, Federowicz S, Lerman JA, Ebrahim A, Palsson BO, Lewis NE (2016) BiGG Models: A platform for integrating, standardizing and sharing genome-scale models. *Nucleic Acids Res* 44: D515-522

Lieven C, Beber ME, Olivier BG, Bergmann FT, Ataman M, Babaei P, Bartell JA, Blank LM, Chauhan S, Correia K *et al* (2020) MEMOTE for standardized genome-scale metabolic model testing. *Nat Biotechnol* 38: 272-276

Ludewig AH, Izrayelit Y, Park D, Malik RU, Zimmermann A, Mahanti P, Fox BW, Bethke A, Doering F, Riddle DL *et al* (2013) Pheromone sensing regulates *Caenorhabditis*

elegans lifespan and stress resistance via the deacetylase SIR-2.1. *Proc Natl Acad Sci U S A* 110: 5522-5527

Mahadevan R, Edwards JS, Doyle FJ, 3rd (2002) Dynamic flux balance analysis of diauxic growth in Escherichia coli. *Biophys J* 83: 1331-1340

McGhee JD (2007) The *C. elegans* intestine. *Wormbook*: www.wormbook.org

Raman K, Chandra N (2009) Flux balance analysis of biological systems: applications and challenges. *Brief Bioinform* 10: 435-449

Sajitz-Hermstein M, Nikoloski Z (2016) Multi-objective shadow prices point at principles of metabolic regulation. *Biosystems* 146: 91-101

Schellenberger J, Park JO, Conrad TM, Palsson BO (2010) BiGG: a Biochemical Genetic and Genomic knowledgebase of large scale metabolic reconstructions. *BMC Bioinformatics* 11: 213

Shlomi T, Cabili MN, Herrgard MJ, Palsson BO, Ruppin E (2008) Network-based prediction of human tissue-specific metabolism. *Nature biotechnology* 26: 1003-1010

Swainston N, Smallbone K, Hefzi H, Dobson PD, Brewer J, Hanscho M, Zielinski DC, Ang KS, Gardiner NJ, Gutierrez JM *et al* (2016) Recon 2.2: from reconstruction to model of human metabolism. *Metabolomics* 12: 109

Uhlen M, Fagerberg L, Hallstrom BM, Lindskog C, Oksvold P, Mardinoglu A, Sivertsson A, Kampf C, Sjostedt E, Asplund A *et al* (2015) Proteomics. Tissue-based map of the human proteome. *Science* 347: 1260419

UniProt C (2019) UniProt: a worldwide hub of protein knowledge. *Nucleic Acids Res* 47: D506-D515

Witting M, Hastings J, Rodriguez N, Joshi CJ, Hattwell JPN, Ebert PR, van Weeghel M, Gao AW, Wakelam MJO, Houtkooper RH *et al* (2018) Modeling Meets Metabolomics-The WormJam Consensus Model as Basis for Metabolic Studies in the Model Organism *Caenorhabditis elegans*. *Front Mol Biosci* 5: 96

Yilmaz LS, Walhout AJ (2016) A *Caenorhabditis elegans g*enome-scale metabolic network model. *Cell Syst* 2: 297-311

Zur H, Ruppin E, Shlomi T (2010) iMAT: an integrative metabolic analysis tool. *Bioinformatics* 26: 3140-3142
