# Supplementary material for: Modeling tissue‐relevant Caenorhabditis elegans metabolism at network, pathway, reaction, and metabolite levels
Source: Mol Syst Biol. 2020 Oct 6;16(10):e9649. doi: 10.15252/msb.20209649 (PMC7537831; doi:10.15252/msb.20209649)
Supplement: Supplementary file 2 — Expanded View Figures PDF [file MSB-16-e9649-s002.pdf]

Expanded View Figures

Figure EV1. Reconstructed ascaroside biosynthesis pathway in iCEL1314.

A Ascaroside biosynthesis from a very long-chain fatty acid (C28) assumed to serve as a precursor for all ascarosides. The unknown pathway of ascarylose synthesis and attachment is represented by two mass- and charge-balanced reactions (RCC0211 and RCC0227, Table EV2). Reactions from the iterative peroxisomal beta-oxidation of long-chain ascaroside are lumped to only keep a minimal number of intermediate metabolites in the reconstruction. In total, the synthesis of seven ascarosides is reconstructed, covering the most abundant ascaroside species in *Caenorhabditis elegans*.

B A detailed representation of the peroxisomal beta-oxidation during ascaroside biosynthesis. The lumped peroxisomal beta-oxidation reactions in (A) represent the net reaction that comes out of one or multiple iterations of the pathway in (B).

© 2020 The Authors

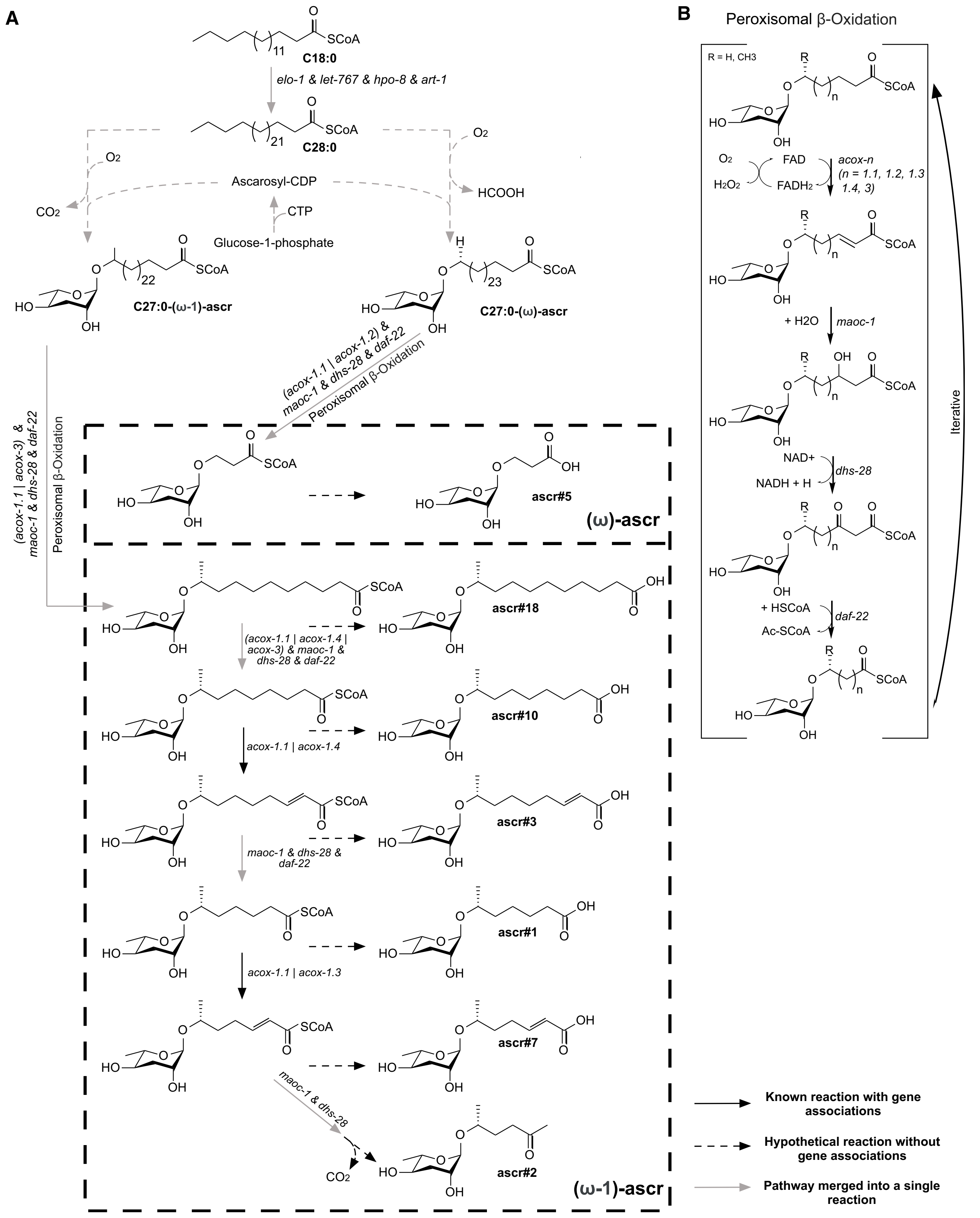

Figure EV1.

© 2020 The Authors

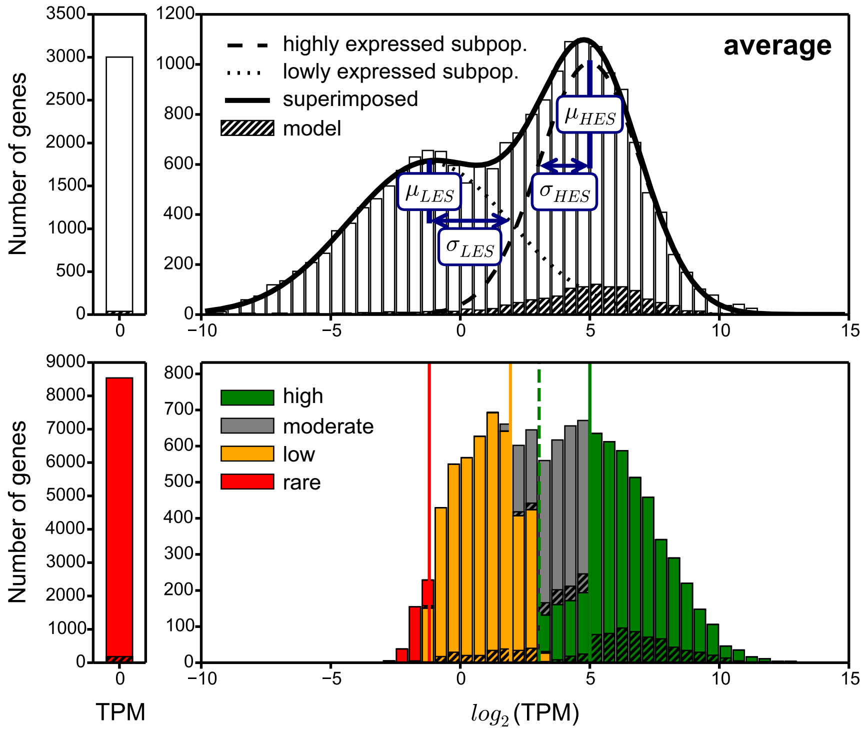

Figure EV2. Determination of expression-level thresholds using gene expression histograms.

Histograms representing average gene expression in seven tissues combined (upper panel) and in the intestine alone as an example tissue (lower panel). The mean (μ) and standard deviation (σ) of high expression (HES) and low expression (LES) subpopulation of genes were determined by curve fitting and used to define the thresholds for gene categorization. Red (μ_{LES}), yellow ($\mu_{LES} + \sigma_{LES}$), and green (μ_{HES}) lines are hard thresholds for rarely, lowly, and highly expressed genes, respectively (lower panel). Dashed green line ($\mu_{HES} + \sigma_{HES}$) indicates the relative expression threshold for describing additional highly and lowly expressed genes based on enrichment/depletion analysis as described in Fig EV3. Bars reflect frequency of genes in the corresponding category based on color. Metabolic genes that are part of iCEL1314 are indicated with hatched bars (see Appendix Fig S1 for the histograms of metabolic genes).

© 2020 The Authors

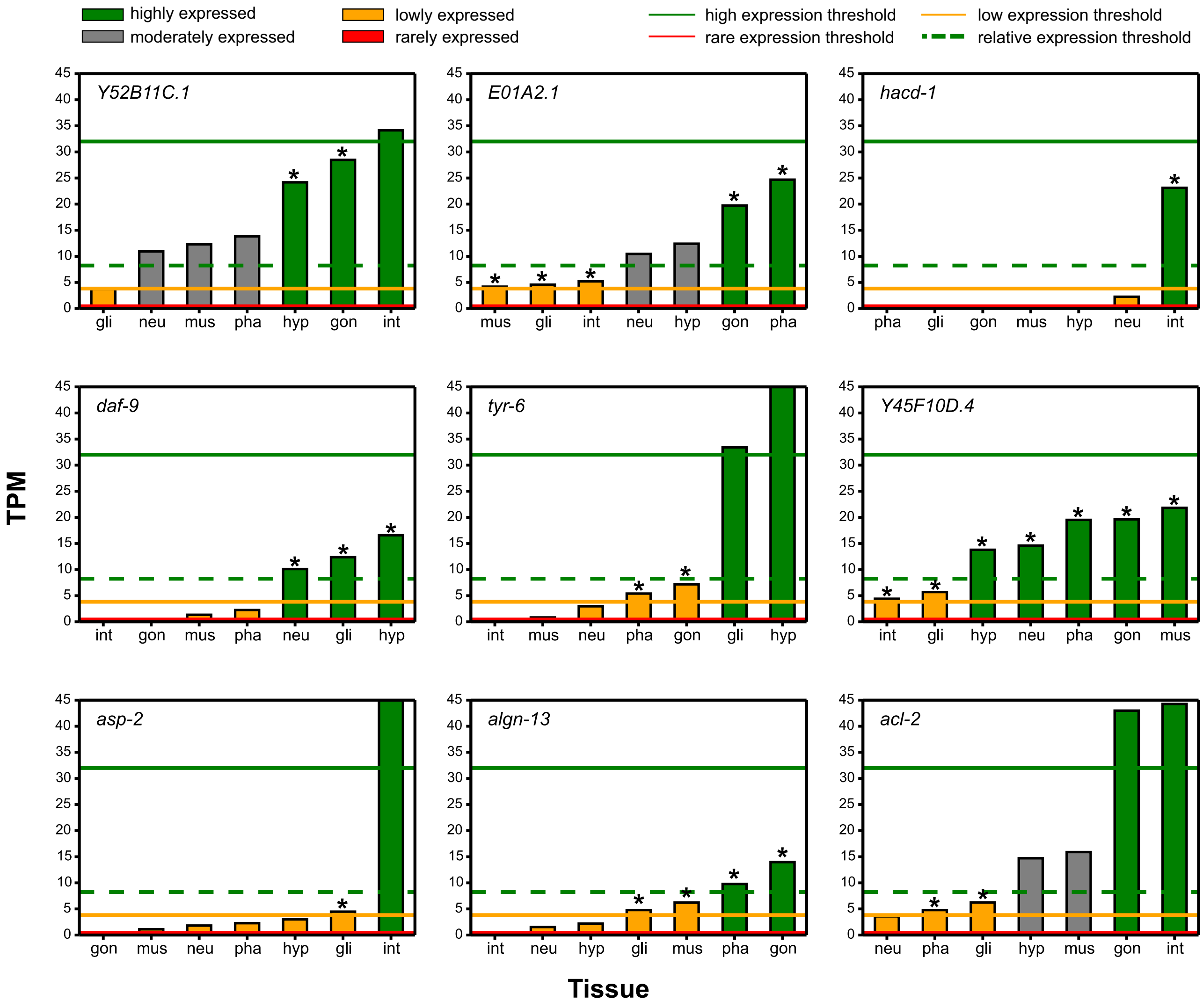

Figure EV3. Modifications in gene expression categorization based on the relative expression analysis algorithm.

Genes that were initially categorized as moderately expressed in some tissues were reevaluated based on a relative expression algorithm (see Appendix Supplementary Methods for details). If a gene is enriched in a tissue and has an expression value that is higher than the relative expression threshold (dashed green line) but lower than the absolute high expression threshold (solid green line), then its category for that tissue was changed from moderately expressed to highly expressed. Conversely, if a gene is depleted in a tissue and has an expression value that is lower than the relative expression threshold but higher than the absolute low expression threshold (yellow line), then its category for that tissue was changed from moderately expressed to lowly expressed. Tissue expression profiles of nine genes are shown as examples. Asterisk (*) indicates values for which the expression category was changed.

© 2020 The Authors

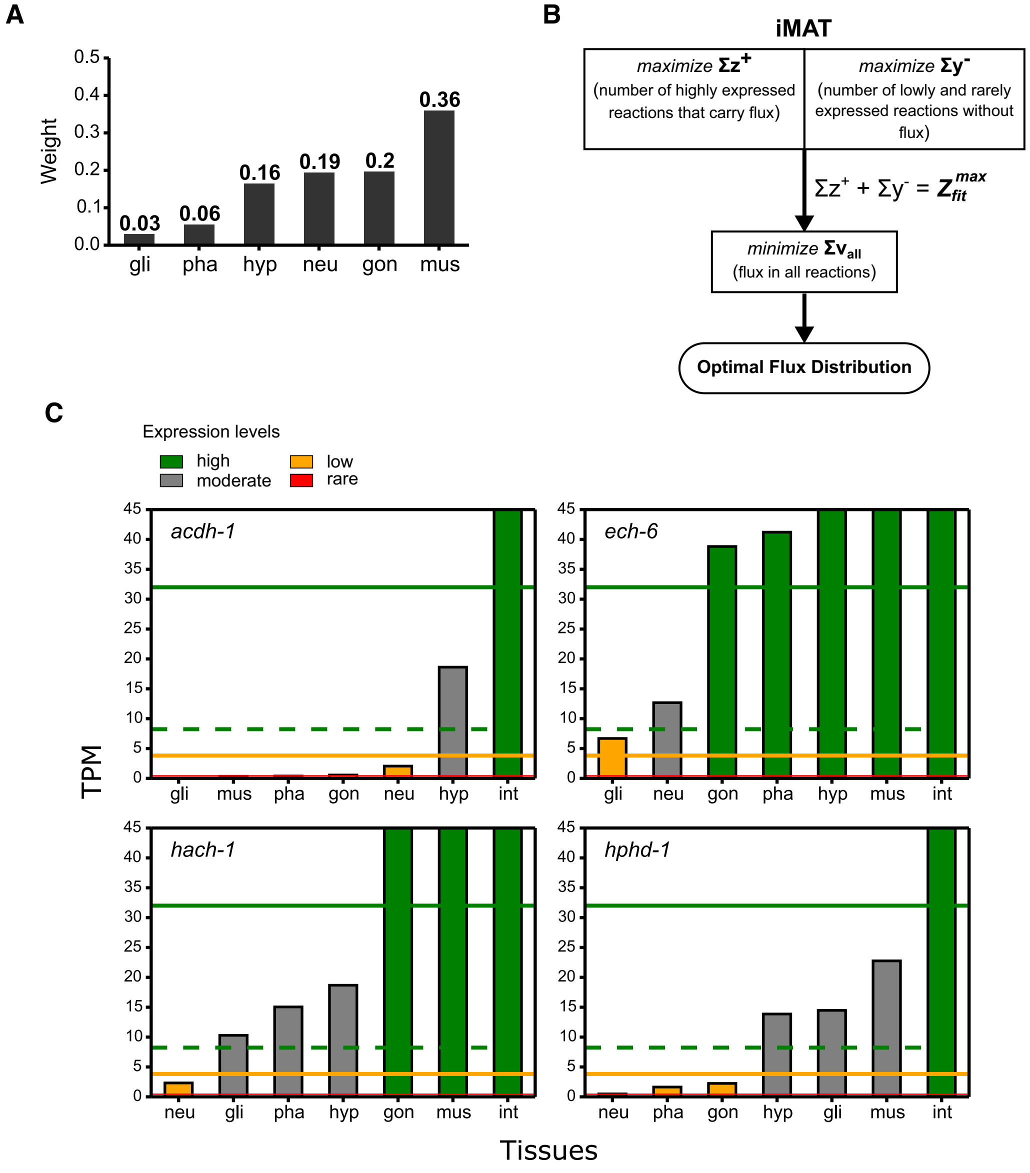

Figure EV4. Additional information on the integration of gene expression data with the dual-tissue model.

A Tissue weights used to calculate flux demands of non-intestinal tissues during the integration of intestine data with the dual-tissue model. The weight of a tissue represents its relative mass or activity according to Appendix Supplementary Methods, Appendix Equation S33. A combined flux distribution of the six tissues was obtained as the product sum of flux distributions (flux vectors of all reactions in the *X* compartment of the dual-tissue model) and weights. Transport fluxes in this combined flux distribution were used during intestine integration (see Appendix Supplementary Methods for details).

B The original integration algorithm (iMAT) (Shlomi *et al*, 2008; Zur *et al*, 2010; Yilmaz & Walhout, 2016), with modifications from Yilmaz and Walhout (2016), is shown as a flowchart as in Fig 2B.

C Expression profiles of genes associated with the partial propionyl-CoA degradation pathway used in Fig 2C. Due to the scale, the rare expression threshold line (solid red) and bars with heights < 0.7 TPM are not immediately visible.

© 2020 The Authors

Figure EV5. Sensitivity analyses with MERGE.

A Sensitivity of key findings (Fig 7) to changes in parameters and methods (see Appendix Supplementary Methods for details). Columns indicate the changes tested, which are indicated on the upper right corner. Rows represent 73 reactions selected from Tables EV7 and EV8 for a particular metabolic function. Row labels indicate, from left to right, the tissue which was found to be a primary or secondary site for the function (see main text), the reaction whose flux represents the function (with directionality shown with the last letter), and (in parentheses) whether the prediction is at the reaction level (rxn; reaction can be found in Table EV7) or metabolite level (met; reaction can be found in Table EV8). Only a subset of reactions was included for related functions with repeated patterns, such as the production of different ascarosides.

B Dendrogram showing the similarity of results with regard to which tissues are matched to which function as primary or secondary sites with high confidence. Matching a function to a tissue in either of these two categories was considered the same, and the Jaccard index was used to determine the ratio of the difference between lists of matchings from different tests. As a reference point, the original dataset has a list of 1,579 high-confidence matchings of functions to primary or secondary tissues.

C Dendrogram showing the similarity of results from different tests based on the cosine angle of vectors that represent relative flux potentials calculated for every tissue at every applicable reaction.

See Appendix Supplementary Methods for the no relative expression analysis test, which is not discussed in the main text.

 © 2020 The Authors

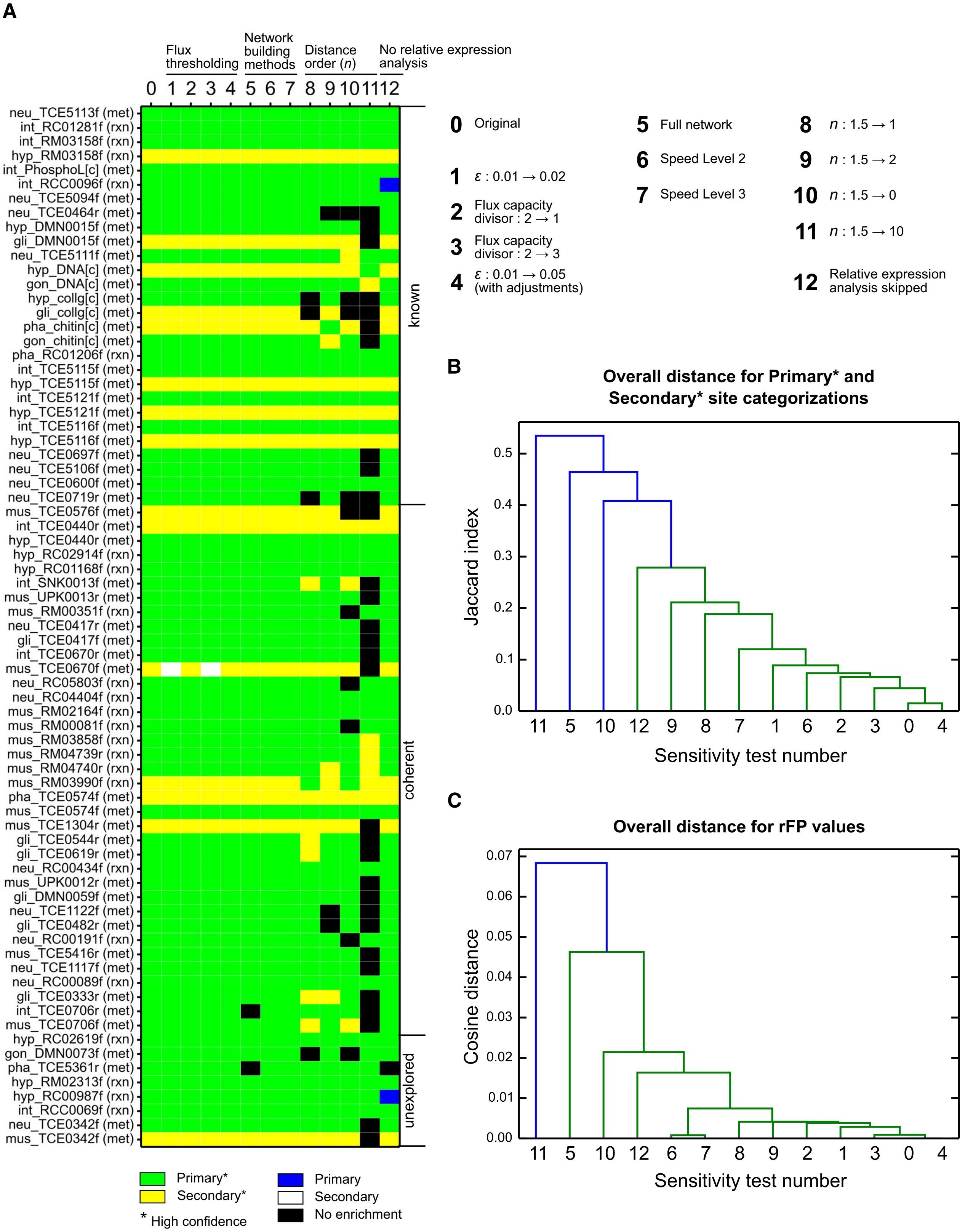
A
Flux thresholding
Network building methods
Distance order (n)
No relative expression analysis
0 Original
1 ε : 0.01 → 0.02
2 Flux capacity divisor : 2 → 1
3 Flux capacity divisor : 2 → 3
4 ε : 0.01 → 0.05 (with adjustments)
5 Full network
6 Speed Level 2
7 Speed Level 3
8 n : 1.5 → 1
9 n : 1.5 → 2
10 n : 1.5 → 0
11 n : 1.5 → 10
12 Relative expression analysis skipped
known
coherent
unexplored
Primary*
Secondary*
* High confidence
Primary
Secondary
No enrichment
B
Overall distance for Primary* and Secondary* site categorizations
Jaccard index
Sensitivity test number
C
Overall distance for rFP values
Cosine distance
Sensitivity test number

Figure EV5.
